# Genomic Characterization of *Cronobacter* spp. and *Salmonella* spp. Strains Isolated From Powdered Infant Formula in Chile

**DOI:** 10.3389/fmicb.2022.884721

**Published:** 2022-06-02

**Authors:** Julio Parra-Flores, Ondřej Holý, Sergio Acuña, Sarah Lepuschitz, Ariane Pietzka, Alejandra Contreras-Fernández, Pamela Chavarría-Sepulveda, Ariadnna Cruz-Córdova, Juan Xicohtencatl-Cortes, Jetsi Mancilla-Rojano, Alejandro Castillo, Werner Ruppitsch, Stephen Forsythe

**Affiliations:** ^1^Department of Nutrition and Public Health, Universidad del Bío-Bío, Chillán, Chile; ^2^Science and Research Centre, Faculty of Health Sciences, Palacký University Olomouc, Olomouc, Czechia; ^3^Department of Food Engineering, Universidad del Bío-Bío, Chillán, Chile; ^4^Austrian Agency for Health and Food Safety, Institute for Medical Microbiology and Hygiene, Vienna, Austria; ^5^Food Quality Testing and Certification Laboratory, Universidad del Bío-Bío, Chillán, Chile; ^6^Intestinal Bacteriology Research Laboratory, Hospital Infantil de México Federico Gómez, Mexico City, Mexico; ^7^Faculty of Medicine, Biological Sciences Graduate Program, Universidad Nacional Autónoma de México, Mexico City, Mexico; ^8^Department of Nutrition and Food Science, Texas A&M University, College Station, TX, United States; ^9^Foodmicrobe.com, Nottingham, United Kingdom

**Keywords:** *Cronobacter sakazakii*, *Cronobacter malonaticus*, *Salmonella* Typhimurium, powdered infant formula, virulence, resistance genes, whole-genome sequencing, CRISPR-Cas

## Abstract

This study characterized five *Cronobacter* spp. and six *Salmonella* spp. strains that had been isolated from 155 samples of powdered infant formula (PIF) sold in Chile and manufactured in Chile and Mexico in 2018–2020. Two strains of *Cronobacter sakazakii* sequence type (ST) ST1 and ST31 (serotypes O:1 and O:2) and one strain of *Cronobacter malonaticus* ST60 (O:1) were identified. All *Salmonella* strains were identified as *Salmonella* Typhimurium ST19 (serotype O:4) by average nucleotide identity, ribosomal multilocus sequence typing (rMLST), and core genome MLST (cgMLST). The *C. sakazakii* and *C. malonaticus* isolates were resistant to cephalothin, whereas the *Salmonella* isolates were resistant to oxacillin and ampicillin. Nineteen antibiotic resistance genes were detected in the *C. sakazakii* and *C. malonaticus* isolates; the most prevalent were mcr-9.1, *bla_CSA_*, and *bla_CMA_*. In *Salmonella*, 30 genes encoding for aminoglycoside and cephalosporin resistance were identified, including *aac(6′)-Iaa*, β-*lactamases ampH*, *ampC1*, and *marA*. In the *Cronobacter* isolates, 32 virulence-associated genes were detected by WGS and clustered as flagellar proteins, outer membrane proteins, chemotaxis, hemolysins, invasion, plasminogen activator, colonization, transcriptional regulator, survival in macrophages, use of sialic acid, and toxin-antitoxin genes. In the *Salmonella* strains, 120 virulence associated genes were detected, adherence, magnesium uptake, resistance to antimicrobial peptides, secretion system, stress protein, toxin, resistance to complement killing, and eight pathogenicity islands. The *C. sakazakii* and *C. malonaticus* strains harbored I-E and I-F CRISPR-Cas systems and carried Col(pHHAD28) and IncFIB(pCTU1) plasmids, respectively. The *Salmonella* strains harbored type I-E CRISPR-Cas systems and carried IncFII(S) plasmids. The presence of *C. sakazakii* and *Salmonella* in PIF is a health risk for infants aged less than 6 months. For this reason, sanitary practices should be reinforced for its production and retail surveillance.

## Introduction

The need to ensure the safety of powdered infant formula (PIF) led the FAO/WHO to establish the microbiological or epidemiological relationship of microbial agents found in PIF with infant infection. They identified three categories of microorganisms based on evidence of a causal relationship between the presence of these microorganisms and the disease they cause. The first category of microorganisms with clear causality were identified as *Cronobacter* spp. and *Salmonella enterica*. The second consists of microorganisms for which causality is possible but has not yet been demonstrated. These were mainly from the *Enterobacteriaceae* family, but also included *Acinetobacter*. Finally, the third involves microorganisms for which causality is less likely or has not yet been shown and has not been identified in PIF. Based on this the FAO/WHO recommended the absence of *Cronobacter* spp. and *Salmonella* in PIF for target age less than 6 months (FAO/WHO, 2004, 2006; Forsythe, 2018).

*Cronobacter* is a genus of bacterial pathogens consisting of seven species: *C. sakazakii*, *C. malonaticus*, *C. universalis*, *C. turicensis*, *C. muytjensii*, *C. dublinensis*, and *C. condimenti* (Iversen et al., 2008; Joseph et al., 2012; Stephan et al., 2014). The species with the greatest clinical significance are *C. sakazakii* and *C. malonaticus* and have been reported in cases and outbreaks associated with PIF in infants (Forsythe, 2018; Parra-Flores et al., 2021a). At present in the United States, three cases of illness caused by PIF contaminated with *C. sakazakii*, resulting in one fatality, and one case of *Salmonella* Newport are being investigated; this has prompted an international voluntary recall of these PIFs by the manufacture (U.S FDA, 2022). The severity of the clinical condition has been associated with the presence of virulence factors encoded on plasmids (Shi et al., 2018; Aly et al., 2019), adherence and invasion traits (Cruz et al., 2011; Parra-Flores et al., 2018a; Holý et al., 2021), and various other genes such as *aut*, *cpA*, *fliC*, *hly*, *ompA*, *sip*, *plas*, and *inv* (Cruz et al., 2011; Franco et al., 2011; Aldubyan et al., 2017; Holý et al., 2019). Other factors are the use of sialic acid as a carbon source, capsule composition and the presence of its capsule, and endotoxin production (Ogrodzki and Forsythe, 2015). Another important aspect is the resistance to β-lactam antibiotics such as cephalothin, cefotaxime, ceftazidime, and ampicillin in addition to the presence of resistance genes such as *marA*, *glpT*, *ampH*, *blaCSA*, and *mcr* (Flores et al., 2011; Lee et al., 2012; Fei et al., 2017; Holý et al., 2021).

*Salmonella enterica* is a gram-negative, rod-shaped, facultative anaerobic genus. More than 2,600 serotypes belonging to *S. enterica* have been described worldwide que incluyen, which can cause diseases in humans and animals (Mezal et al., 2014). *Salmonella* is the most widely studied microbial pathogen, and can be isolated from a variety of foods, including PIF associated with disease outbreaks in infants (Angulo et al., 2008; Carrasco et al., 2012; Jourdan-da Silva et al., 2018). Gastroenteric *Salmonella* infections usual develop as self-limiting gastroenteritis, and antibiotic treatment is necessary only in severe cases more often associated with immunocompromised patients or those at the extremes of age such as infants (de Toro et al., 2014). Therefore, the emergence of strains that are resistant to β-lactams and cephalosporins is a relevant public health and food safety problem (Güerri et al., 2004; de Toro et al., 2011; Wang et al., 2019). In addition, *Salmonella* exhibits virulence factors that play a decisive role in systemic infections, such as pathogenicity islands (PAIs), invasion and adherence genes, and enterotoxin coding (Murugkar et al., 2003; Nayak et al., 2004; Huehn et al., 2010; Thung et al., 2018).

*Salmonella* and *Cronobacter* species are known persist in low-moisture foods such as milk powder and powdered infant formula for up to 2 years (Caubilla-Barron and Forsythe, 2007). Consequently, outbreaks due to the consumption of contaminated products have been reported (Forsythe, 2018; Jones et al., 2019). *Cronobacter* can survive spray-drying and persist in the manufacturing environment as biofilms. Genotyping has shown the persistence of specific *Salmonella* and *Cronobacter* strains within production facilities for many years (Craven et al., 2010; Jones et al., 2019). Contamination may occur post-pasteurization due to the addition of contaminated ingredients (FAO/WHO, 2008).

Whole-genome sequencing (WGS) has facilitated the in-depth study of pathogenic organisms by generating extensive information that helps to determine relationships and taxonomic differences between them (Leopold et al., 2014). It is not only used for isolate identification, but also extensive profiling and genotyping; such as conventional 7-loci multilocus sequence typing (MLST), core genome MLST (cgMLST) and/or single nucleotide polymorphism (SNP) analysis, molecular serotyping, CRISPR-Cas array profiling, and detection of genes associated with antibiotic resistance and virulence. Consequently, more precise epidemiological links can be established (Marraffini, 2013; Leopold et al., 2014; Uelze et al., 2020). Therefore, the analysis of the complete genomes and their comparison enables a more complete analysis of the pathogenesis process of *C. sakazakii* (Lehner et al., 2018).

In 2017, a recall of powdered formula samples contaminated with *Cronobacter* occurred in Chile (Parra-Flores et al., 2018b). This situation led to the incorporation of microbial criteria (*n* = 30; *c* = 0) for *Cronobacter* spp. in PIF intended for consumption by infants aged less than 12 months into the Chilean Food Sanitary Regulations (RSA; Parra-Flores et al., 2018b), given that the microbiological criteria in the RSA for Salmonella was already defined (*n* = 10; *c* = 0). This study considers the safety of PIF from 2018 to 2020 with the objective of performing a genomic characterization of five *Cronobacter* spp. and six *Salmonella* strains isolated from PIF sold in Chile. These PIF had been and manufactured in Chile and Mexico.

## Materials and Methods

### Sampling

A total of 155 PIF samples from two commercial brands whose main ingredient was casein and whey were analyzed. Of these, 80 PIF samples were made in Chile and 75 PIF samples were made in Mexico. The experimental units, milk cans, were obtained monthly from supermarkets and pharmacies because these products are replenished monthly. In addition, this allowed obtaining greater variability in terms of the origin of the production batch.

### Isolation and Identification

*Cronobacter* were isolated according to the method described by Iversen and Forsythe (2004). For each sample, 225 ml buffered peptone water (BPW) was added to 25 g PIF, homogenized in a stomacher at a mean velocity for 60 s, and incubated at 37°C. For *Cronobacter* spp., 10 ml of each sample was inoculated after incubation at 37°C for 24 h in 90 ml *Enterobacteriaceae* enrichment broth (BD Difco, Sparks, MD, United States). A loop was extracted from the culture suspension and striated in Brilliance Chromogenic Agar CM 1035 (OxoidTermo-Fisher, Hampshire, United Kingdom) at 37°C for 20 h. Five strains, presumed to be colonies of *Cronobacter* spp. (green or blue), were striated in trypticase soy agar (BD Difco, Sparks, MD, United States) to verify their purity prior to future analyses. The isolated strains were maintained in a strain collection and stored at −80°C.

The official method (Instituto Nacional de Normalización, 2002) NCh 2675- ISO 6572-2 rev 2017 for *Salmonella* in Chile was used. From the initial incubated sample of 25 g PIF with 225 ml BPW for 24 h, 0.1 ml was inoculated in 10 ml of Rappaport-Vassiliadis with soya broth (RVS, Oxoid, Hampshire, United Kingdom) and 1 ml in Muller-Kauffmann-Tetrathionate-Novobiocin broth (MKTTn, Merck, Darmstadt, Germany) incubated for 24 ± 3 h at 41.5°C and 37°C, respectively. The colonies were then isolated in Xylose Lysine Deoxycoholate (XLD, Merck, Darmstadt, Germany) and Salmonella chromogenic agar incubated at 37°C for 24 h. The typical colonies were confirmed by biochemical tests. Both pathogens were identified by Matrix-Assisted Laser Desorption Ionization Time of Flight Mass Spectrometry (MALDI-TOF MS; Bruker, Billerica, MA, United States) and with the MBT Compass IVD software 4.1.60 (Bruker) described by Lepuschitz et al. (2017).

### Whole-Genome Sequencing

Before WGS, all the *Cronobacter* spp. and *Salmonella* spp. strains were cultured in Columbia blood agar plates (bioMérieux, Marcy-l’Étoile, France) at 37°C for 24 h. DNA was isolated from bacterial cultures with the MagAttract HMW DNA Kit (Qiagen, Hilden, Germany) according to the manufacturer’s instructions. The amount of DNA was quantified on a Lunatic instrument (Unchained Labs, Pleasanton, CA, United States). Nextera XT chemistry (Illumina Inc., San Diego, CA, United States) was used to prepare sequencing libraries for a 2 × 300 bp paired-end sequencing run on an Illumina MiSeq sequencer. Samples were sequenced to achieve a minimum of 80-fold coverage using standard protocols by Illumina. The resulting FASTQ files were quality trimmed and *de novo* assembled with the SPAdes version 3.9.0. Contigs were filtered for a minimum of 5-fold coverage and 200 bp minimum length with Ridom SeqSphere+ software v. 7.8.0 (Ridom, Münster, Germany; Jünemann et al., 2013).

### Sequence Type and Core Genome Multilocus Sequence Typing of *Cronobacter* spp. and *Salmonella* spp.

A total of 3,678 targets were used to establish the core genome multilocus sequence typing (cgMLST) scheme of *Cronobacter* spp. using strain ATCC BAA-894 as a reference using Ridom SeqSphere+ software v. 7.8.0 (Ridom, Münster, Germany; Jünemann et al., 2013). For *Salmonella*, the cgMLST scheme was performed based on the profile of 2,969 *S. enterica* target gene loci task template of the Ridom SeqSphere+ software v. 7.8.0 (Ridom, Münster, Germany). According to the cgMLST scheme, isolates were visualized with a minimum spanning tree (MST) to establish their genotypic relationships (Lepuschitz et al., 2019). In addition, the sequences of the seven housekeeping genes of the conventional MLST for *Cronobacter* spp. and *Salmonella* were extracted and cross-checked against the *Cronobacter* MLST database^1^ (Baldwin et al., 2009) and *Salmonella* MLST^2^ (Achtman et al., 2012), respectively. The *Cronobacter* strains are ID 3409–3413 in the *Cronobacter* PubMLST database and *Salmonella* are ID RID389119–RID389124 in the cgMLST database.

### Determination of Serotypes

The *gnd* and *galF* genes that are specific to the *Cronobacter* serotype O region was determined by WGS sequence analysis with the BIGSdb tool available in the PubMLST database^3^ and CroTrait WGS analysis (Wang L. et al., 2021). For *Salmonella*, the SeqSero 1.2 tool available at https://cge.cbs.dtu.dk/services/SeqSero/ was used (Zhang et al., 2015).

### Antibiotic Resistance Profile

The disk diffusion method was used in accordance with the recommendations of the Clinical and Laboratory Standards Institute (CLSI, 2020). The commercial disks that were used consist of ampicillin (10 μg), amikacin (30 μg), cephalothin (30 μg), chloramphenicol (30 μg), ceftriaxone (30 μg), cefotaxime (30 μg), cefepime (30 μg), gentamicin (10 μg), levofloxacin (5 μg), netilmicin (30 μg), oxacillin (1 μg), and sulfamethoxazole-trimethoprim (1.25/23.75 μg). The characterization of the resistance/susceptibility profiles was determined according to the CLSI guidelines. The *Escherichia coli* ATCC 25922 and *Pseudomonas aeruginosa* ATCC 27853 strains were used as references.

### Detection of Antibiotic Resistance and Virulence Genes

The existence of virulence genes was confirmed by applying the task template function in SeqSphere+ for the WGS data and the ResFinder tool from the Center of Genomic Epidemiology (CGE).^4^ Thresholds for the target scanning procedure were set with a required identity of ≥90% to the reference sequence and an aligned reference sequence ≥99%. The Comprehensive Antibiotic Resistance Database (CARD) with the “perfect” and “strict” default settings for sequence analysis (Jia et al., 2017), the Task Template AMRFinderPlus 3.2.3 available in Ridom SeqSphere+ v. 7.8.0 software using the EXACT method at 100%, and BLAST alignment for protein identification available in the AMRFinderPlus database were used for antimicrobial resistance genes.

### Detection of Plasmids and Mobile Genetic Elements

The PlasmidFinder 2.1 and MobileElementFinder 1.0 tools were used to detect plasmids and mobile genetic elements (MGEs). The selected minimum identity was 95% and 90%, respectively (Carattoli et al., 2014; Johansson et al., 2021).^4^

### Profiling of CRISPR-Cas Loci Profiling

The search and characterization of CRISPR arrays and their association with Cas proteins was determined with CRISPRDetect and CRISPRminer (available at http://crispr.otago.ac.nz/CRISPRDetect/predict_crispr_array.html and http://www.microbiome-bigdata.com/CRISPRminer; Biswas et al., 2016; Zhang et al., 2018). The following parameters were applied: 18–55 pb repeated sequence length, 25–60 pb spacer length, 0.6–2.5 spacer sequence size as a function of repeated sequence size, and 60% maximum percentage similarity between spacers. The PHASTER program (available at https://phaster.ca/) was used to identify sequences associated with prophages within the study genomes, and the phages associated with the spacer sequences were determined with the CRISPRminer program. The types of CRISPR systems were determined with the CRISPRmap program (Lange et al., 2013). The CRISPRTarget program was used to determine the PAM (protospacer adjacent motif) sequences associated with each of the repeated sequences of the identified arrays.

## Results

### Identification, Genotyping, and Antibiotic Resistance Profiles of *Cronobacter* and *Salmonella* Isolates

Overall positivity for the *Cronobacter* spp. samples was 6.25% (5/80) and 2.7% (2/75) for *Salmonella*. Of the five *Cronobacter* spp. presumptive strains, four were identified as *C. sakazakii* and one as *C. malonaticus*. All strains were isolated from different PIF batches from the same manufacturer and country (Chile). The six *Salmonella* strains were identified as *Salmonella* Typhimurium. These were from two different batches and tins but same manufacturer and country (Mexico; Table 1).

**Table 1:** Identification of *Cronobacter* spp. and *Salmonella* spp. strains isolated from powdered infant formula by matrix-assisted laser desorption ionization time-of-flight mass spectrometry (MALDI-TOF MS) and whole-genome sequencing (WGS).

Sample ID (MLST database)	Country	MALDI-TOF MS	WGS	ST	CC	Serotype
510197-19 (^*^3409)	Chile	*C. sakazakii*	*C. sakazakii*	1	1	O-1
510199-19 (^*^3410)	Chile	*C. sakazakii*	*C. sakazakii*	1	1	O-1
510290-19 (^*^3411)	Chile	*C. sakazakii*	*C. sakazakii*	1	1	O-1
510556-19 (^*^3412)	Chile	*C. sakazakii*	*C. sakazakii*	31	31	O-2
510557-19 (^*^3413)	Chile	*C. malonaticus*	*C. malonaticus*	60	60	O-1
510535-21 (^**^RD389119)	Mexico	*S.* Typhimurium	*S.* Typhimurium	19	19	O-4:-
510536-21 (^**^RD389120)	Mexico	*S.* Typhimurium	*S.* Typhimurium	19	19	O-4:-:-
510537-21 (^**^RD389121)	Mexico	*S.* Typhimurium	*S.* Typhimurium	19	19	O-4:i:1,2
510538-21 (^**^RD389122)	Mexico	*S.* Typhimurium	*S.* Typhimurium	19	19	O:4:i:1,2
510539-21 (^**^RD389123)	Mexico	*S.* Typhimurium	*S.* Typhimurium	19	19	^*^O-4:i:-
510540-21 (^**^RD389124)	Mexico	*S.* Typhimurium	*S.* Typhimurium	19	19	^*^O-4:i:-

ST, sequence type and CC, clonal complex.

* MLST database ID.

** cgMLST database ID. * Potential monophasic variant of S. Typhimurium.

**Figure 1:**
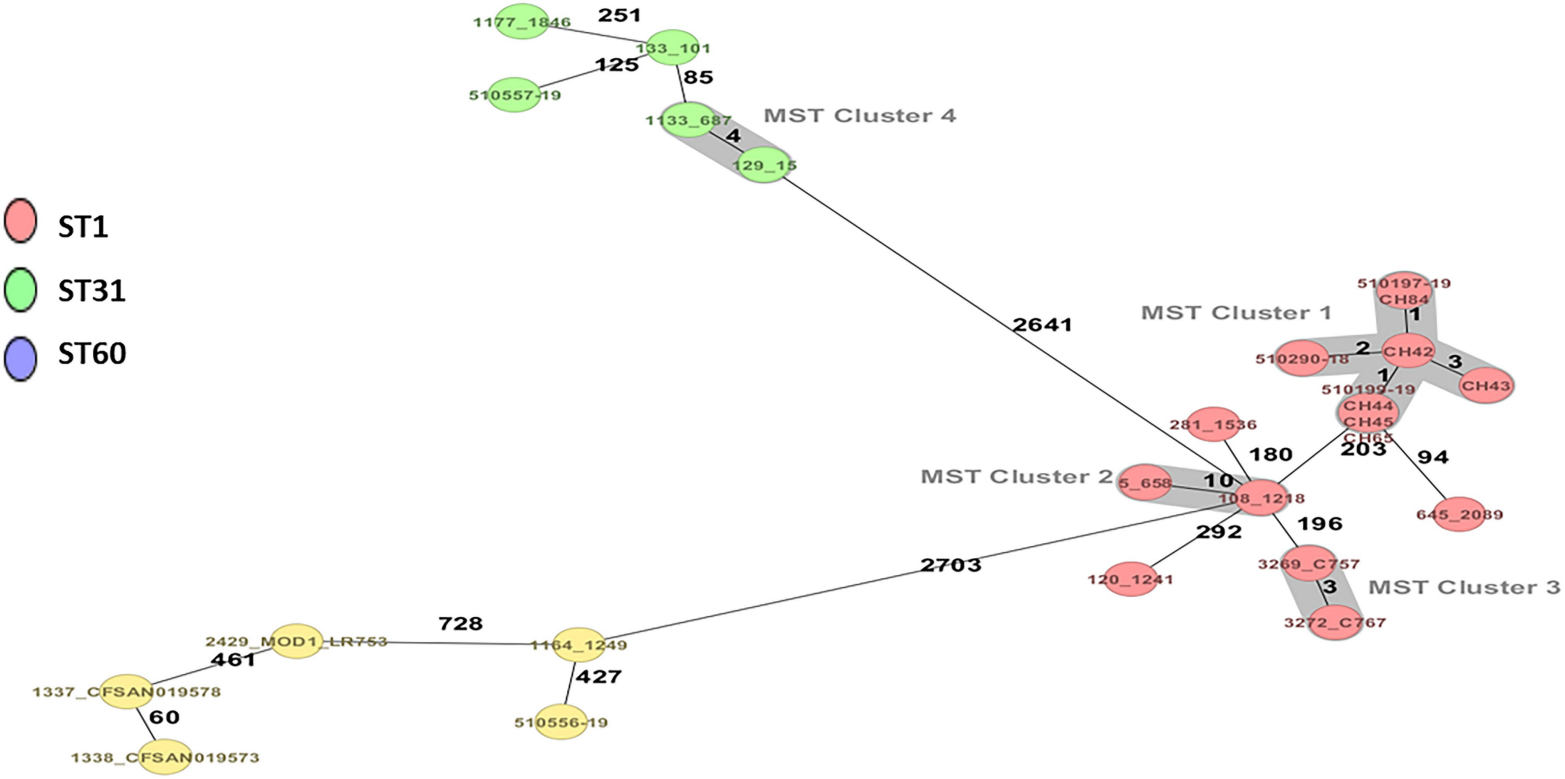
Minimum spanning tree (MST) of five strains of *Cronobacter sakazakii* and one of *Cronobacter malonaticus* from powdered infant formula manufactured in Chile, complemented with strains of *C. sakazakii* and *C. malonaticus* ST1, ST31, and ST60 of clinical and food origin. Calculation of the MST was based on the defined cgMLST scheme comprising 3,678 target genes for *C. sakazakii* and *C. malonaticus.* Isolates are represented as colored circles according to the classical MLST. Black numbers according to the allelic differences between isolates. Isolates with closely related genotypes are marked as Cluster.

Three strains of *C. sakazakii* ST1 (CC1) and ST31, CC31 (serotypes *Csak*: O:1 and O:2, respectively), and one strain of *C. malonaticus* ST60, CC60 (O:1) were identified by average nucleotide identity, rMLST, and cgMLST (Figure 1; Table 1).

**Figure 2:**
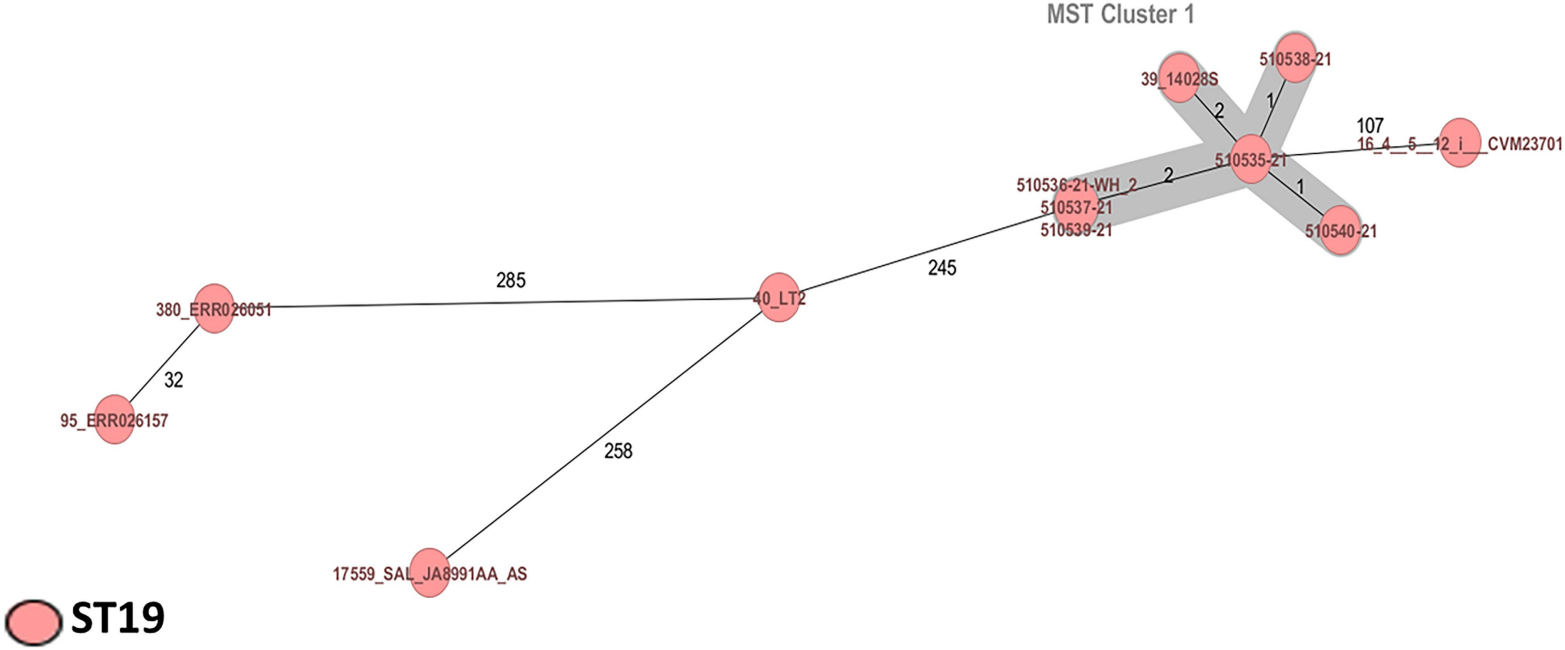
Minimum spanning tree of six *Salmonella* Typhimurium strains from powdered infant formula manufactured in Mexico and supplemented with other *S*. Typhimurium ST19 strains of clinical and food origin. Calculation of the MST was based on the defined cgMLST scheme comprising 3,002 target genes for *Salmonella.* Isolates are represented as colored circles according to the classical MLST. Black numbers according to the allelic differences between isolates. Isolates with closely related genotypes are marked as Cluster.

All *Salmonella* strains were identified as *S.* Typhimurium ST19 (CC19; serotype O:4). Two *Salmonella* strains (510539-21 and 510540-21) were identified as potential monophasic variants of *S.* Typhimurium according to SeqSero analysis (Figure 2; Table 1).

All the *C. sakazakii* and *C. malonaticus* strains were susceptible to 10 of the 12 evaluated antibiotics. However, 100% of the *Cronobacter* strains were resistant to cephalothin and 40% to ampicillin. Meanwhile, 100% of the *Salmonella* isolates were resistant to oxacillin, 83% to ampicillin, 66.6% to cephalothin, and 16.6% to gentamicin (Table 2).

**Table 2:** Antibiotic resistance profile of *Cronobacter* spp. and *Salmonella* spp. strains.

	Strain ID	Species	Antibiotics											
			AM (10 μg)	AK (30 μg)	CL (30 μg)	CRO (30 μg)	CTX (30 μg)	FEP (30 μg)	GE (10 μg)	KF (30 μg)	LEV (5 μg)	NET (30 μg)	OX (1 μg)	SXT (25 μg)
*Cronobacter* spp.	510197-19	*C. sakazakii*	**R**	S	S	S	S	S	S	**R**	S	S	S	S
	510199-19	*C. sakazakii*	S	S	S	S	S	S	S	**R**	S	S	S	S
	510290-19	*C. sakazakii*	S	**S**	S	S	S	S	S	**R**	S	S	S	S
	510556-19	*C. sakazakii*	I	**S**	S	S	S	S	S	**R**	S	S	S	S
	510557-19	*C. malonaticus*	**R**	S	S	S	S	S	S	**R**	S	S	S	S
*Salmonella* spp.	510535-21	*S.* Typhimurium	I	S	S	S	S	S	I	S	S	S	**R**	S
	510536-21	*S.* Typhimurium	**R**	**S**	S	S	I	S	**R**	S	S	S	**R**	S
	510537-21	*S.* Typhimurium	**R**	S	S	S	S	S	S	**R**	S	S	**R**	S
	510538-21	*S.* Typhimurium	**R**	S	S	S	S	S	S	**R**	S	S	**R**	S
	510539-21	*S.* Typhimurium	**R**	S	S	S	S	S	S	**R**	S	S	**R**	S
	510540-21	*S.* Typhimurium	**R**	S	S	S	S	S	S	**R**	S	S	**R**	S

AM, ampicillin; AK, amikacin; CL, chloramphenicol; CRO, ceftriaxone; CTX, cefotaxime; FEP, cefepime; GE, gentamicin; KF, cephalothin; LEV, levofloxacin; NET, netilmicin; OX, oxacillin; SXT, trimethoprim/sulfamethoxazole; **R**, resistance; and **S**, susceptibility; **I**, intermediate. The values in parentheses in bold correspond to the concentrations of antibiotics.

### Detection of Antibiotic Resistance and Virulence Genes

A total of 19 antibiotic resistance genes were detected in the *C. sakazakii* and *C. malonaticus* isolates. All the *C. sakazakii* exhibited *bla*_CSA-1_ and the *C. malonaticus* strain showed *bla*_CMA-1_, conferring resistance to cephalosporins. Both *C. sakazakii* ST1 strains harbored the *mcr-9.1* gene, conferring resistance to colistin. All the *C. sakazakii* and *C. malonaticus* strains exhibited the same efflux genes (*adeF*, *H-NS*, *msbA*, *marA*, *kpnF*, *kpnE*, *emrR*, *emrB*, *rsmA*, and *CRP*), antibiotic inactivation gene (*ampH*), and four antibiotic target alteration genes (*pBP3*, *glpT*, *eF-Tu*, and *marR*; Table 3).

**Table 3:** Antibiotic-resistant genes of *Cronobacter* spp. strains identified by Comprehensive Antibiotic Resistance Database (CARD).

Best hits antibiotic resistance ontology (ARO)	Drug class	Resistance mechanism	510197-19 (ST1)	510199-19 (ST1)	510290-18 (ST1)	510556-19 (ST31)	510557-19 (ST60)
*MCR-9.1*	Peptide antibiotic	Antibiotic target alteration	+	+	+	−	−
*CSA-1*	Cephalosporin	Antibiotic inactivation	+	+	+	+	−
*CMA-1*	Cephalosporin	Antibiotic inactivation	−	−	−	−	+
*pBP3*	Cephalosporin, cephamycin, and penam	Antibiotic target alteration	+	+	+	+	+
*glpT*	Fosfomycin	Antibiotic target alteration	+	+	+	+	+
*eF-Tu*	Elfamycin antibiotic	Antibiotic target alteration	+	+	+	+	+
*marR*	Fluoroquinolone antibiotic, triclosan, rifamycin antibiotic, penam, phenicol antibiotic, glycylcycline, tetracycline antibiotic, and cephalosporin	Antibiotic target alteration	+	+	+	+	+
*adeF*	Fluoroquinolone antibiotic and tetracycline antibiotic	Antibiotic efflux	+	+	+	+	+
*H-NS*	Macrolide antibiotic, fluoroquinolone antibiotic, cephalosporin, cephamycin, penam, and tetracycline antibiotic	Antibiotic efflux	+	+	+	+	+
*msbA*	Nitroimidazole antibiotic	Antibiotic efflux	+	+	+	+	+
*marA*	Fluoroquinolone antibiotic, monobactam, carbapenem, cephalosporin, glycylcycline, cephamycin, penam, tetracycline antibiotic, rifamycin antibiotic, phenicol antibiotic, triclosan, and penem	Antibiotic efflux	+	+	+	+	+
*pnF*	Macrolide antibiotic, aminoglycoside antibiotic, cephalosporin, tetracycline antibiotic, peptide antibiotic, and rifamycin antibiotic	Antibiotic efflux	+	+	+	+	+
*kpnE*	Macrolide antibiotic, aminoglycoside antibiotic, cephalosporin, tetracycline antibiotic, peptide antibiotic, and rifamycin antibiotic	Antibiotic efflux	+	+	+	+	+
*emrR*	Fluoroquinolone antibiotic	Antibiotic efflux	+	+	+	+	+
*emrB*	Fluoroquinolone antibiotic	Antibiotic efflux	+	+	+	+	+
*rsmA*	Fluoroquinolone antibiotic, diaminopyrimidine antibiotic, and phenicol antibiotic	Antibiotic efflux	+	+	+	+	+
*cRP*	Fluoroquinolone antibiotic, macrolide antibiotic, and penam	Antibiotic efflux	+	+	+	+	+
*kpnH*	Macrolide antibiotic, fluoroquinolone antibiotic, aminoglycoside antibiotic, carbapenem, cephalosporin, penam, peptide antibiotic, and penem	Antibiotic efflux	−	−	−	−	−
*ampH ampC-type* β-*lactamase*	Cephalosporin and penam	Antibiotic inactivation	+	+	+	+	+

+, presence and −, absence.

In *Salmonella*, 30 genes that encode for aminoglycoside and cephalosporin resistance were identified, including *aac(6′)-Iaa*, *ampH*, *ampC1*, and *marA*. All the strains exhibited the same efflux genes (*acrAB*, *golS*, *mdsA*, *adeF*, *marA*, *kpnF*, *kpnE*, *emrRB*, *rsmA*, *baeR*, *H-NS*, *sdiA*, *mdfA*, *mdtK*, and *kdpE*), three antibiotic resistance genes (*aac(6′)-Iaa*, β-*lactamase ampH*, and *ampC1*), and nine antibiotic target alteration genes (*bacA*, *pmrF*, *uhpT*, *glpT*, *PBP3*, *EF-Tu*, *soxS*, *soxR*, and *marR*; Table 4).

**Table 4:** Antibiotic-resistant genes of *S.* Typhimurium strains identified by CARD.

Best hits antibiotic resistance ontology (ARO)	Drug class	Resistance mechanism	510535-21	510536-21	510537-21	510538-21	510539-21	510540-21
*AAC(6′)-Iaa*	Aminoglycoside antibiotic	Antibiotic inactivation	+	+	+	+	+	+
*ampH* β-*lactamase*	Cephalosporin and penam	Antibiotic inactivation	+	+	+	+	+	+
*ampC1* β-*lactamase*	Cephalosporin and penam	Antibiotic inactivation	+	+	+	+	+	+
*bacA*	Peptide antibiotic	Antibiotic target alteration	+	+	+	+	+	+
*pmrF*	Peptide antibiotic	Antibiotic target alteration	+	+	+	+	+	+
*uhpT*	Fosfomycin	Antibiotic target alteration	+	+	+	+	+	+
*glpT*	Fosfomycin	Antibiotic target alteration	+	+	+	+	+	+
*PBP3*	Cephalosporin, cephamycin, and penam	Antibiotic target alteration	+	+	+	+	+	+
*EF-Tu*	Elfamycin antibiotic	Antibiotic target alteration, antibiotic efflux	+	+	+	+	+	+
*soxS*	Fluoroquinolone antibiotic, monobactam, carbapenem, cephalosporin, glycylcycline, cephamycin, penam, tetracycline antibiotic, rifamycin antibiotic, phenicol antibiotic, triclosan, and penem	Antibiotic target alteration, antibiotic efflux, reduced permeability to antibiotic	+	+	+	+	+	+
*soxR*	Fluoroquinolone antibiotic, cephalosporin, glycylcycline, penam, tetracycline antibiotic, rifamycin antibiotic, phenicol antibiotic, and triclosan	Antibiotic target alteration, antibiotic efflux, and reduced permeability to antibiotic	+	+	+	+	+	+
*marR*	Fluoroquinolone antibiotic, cephalosporin, glycylcycline, penam, tetracycline antibiotic, rifamycin antibiotic, phenicol antibiotic, and triclosan	Antibiotic target alteration, antibiotic efflux	+	+	+	+	+	+
*acrA*	Fluoroquinolone antibiotic, cephalosporin, glycylcycline, penam, tetracycline antibiotic, rifamycin antibiotic, phenicol antibiotic, and triclosan	Antibiotic efflux	+	+	+	+	+	+
*AcrB*	Fluoroquinolone antibiotic, cephalosporin, glycylcycline, penam, tetracycline antibiotic, rifamycin antibiotic, phenicol antibiotic, and triclosan	Antibiotic efflux	+	+	+	+	+	+
*golS*	Monobactam, carbapenem, cephalosporin, cephamycin, penam, phenicol antibiotic, and penem	Antibiotic efflux	+	+	+	+	+	+
*MdsA*	Monobactam, carbapenem, cephalosporin, cephamycin, penam, phenicol antibiotic, and penem	Antibiotic efflux	+	+	+	+	+	+
*adeF*	Fluoroquinolone antibiotic, and tetracycline antibiotic	Antibiotic efflux	+	+	+	+	+	+
*marA*	Fluoroquinolone antibiotic, monobactam, carbapenem, cephalosporin, glycylcycline, cephamycin, penam, tetracycline antibiotic, rifamycin antibiotic, phenicol antibiotic, triclosan, and penem	Antibiotic efflux, reduced permeability to antibiotic	+	+	+	+	+	+
*kpnE*	Macrolide antibiotic, aminoglycoside antibiotic, cephalosporin, tetracycline antibiotic, peptide antibiotic, and rifamycin antibiotic	Antibiotic efflux	+	+	+	+	+	+
*kpnF*	Macrolide antibiotic, aminoglycoside antibiotic, cephalosporin, tetracycline antibiotic, peptide antibiotic, and rifamycin antibiotic	Antibiotic efflux	+	+	+	+	+	+
*emrR*	Fluoroquinolone antibiotic	Antibiotic efflux	+	−	+	+	+	+
*emrB*	Fluoroquinolone antibiotic	Antibiotic efflux	+	+	+	+	+	+
*rsmA*	Fluoroquinolone antibiotic, diaminopyrimidine antibiotic, and phenicol antibiotic	Antibiotic efflux	+	+	+	+	+	+
*baeR*	Aminoglycoside antibiotic and aminocoumarin antibiotic	Antibiotic efflux	+	+	+	+	+	+
*H-NS*	Macrolide antibiotic, fluoroquinolone antibiotic, cephalosporin, cephamycin, penam, and tetracycline antibiotic	Antibiotic efflux	+	+	+	+	+	+
*sdiA*	Fluoroquinolone antibiotic, cephalosporin, glycylcycline, penam, tetracycline antibiotic, rifamycin antibiotic, phenicol antibiotic, and triclosan	Antibiotic efflux	+	+	+	+	+	+
*mdfA*	Fluoroquinolone antibiotic, macrolide antibiotic, and penam	Antibiotic efflux	+	+	+	+	+	+
*MdtK*	Fluoroquinolone antibiotic	Antibiotic efflux	+	+	+	+	+	+
*CRP*	Macrolide antibiotic, fluoroquinolone antibiotic, and penam	Antibiotic efflux	−	+	+	+	+	+
*kdpE*	Aminoglycoside antibiotic	Antibiotic efflux	+	+	+	+	+	+

+, presence and −, absence.

*Cronobacter sakazakii* isolates showed 32 virulence genes that were detected by WGS and clustered as flagellar proteins, outer membrane proteins, chemotaxis, hemolysins, invasion, plasminogen activator (*cpa*), colonization, transcriptional regulator, survival in macrophages, utilization of sialic acid (*nanA,K,T*), desiccation tolerance (*cheB*, *wzzB*), and toxin-antitoxin genes (*fic*, *relB*). In the *C. malonaticus* strain, the same virulence genes were detected as found in *C. sakazakii*, except for the *cpa* and *nanAK,T* genes (Table 5).

**Table 5:** Putative virulence and distribution of other genes in seven strains of *Cronobacter* spp. by WGS.

Virulence gene	Function	510197-19 (ST1)	510199-19 (ST1)	510290-18 (ST1)	510556-19 (ST31)	510557-19 (ST60)	*C. sakazakii* BAA-894 (ST1)	*C. malonaticus* LMG23826T (ST7)	ES15 control (ST125)
*flgB*	Motility	+	+	+	+	+	+	+	+
*flgK*	Flagellar hook-associated protein 1	+	+	+	+	+	+	+	+
*flgL*	Flagellar hook-associated protein 3	+	+	+	+	+	+	+	−
*flgM*	Negative regulator of flagellin synthesis	+	+	+	+	+	+	+	+
*flgN*	Flagellar synthesis FlgN protein	+	+	+	+	+	+	+	+
*flhD*	Flagellar hook-associated protein 2	+	+	+	+	+	+	+	+
*fliA*	Flagellar operon FliA	+	+	+	+	+	+	+	+
*fliC*	Flagellin	+	+	+	+	+	−	+	−
*fliD*	Flagellar hook-associated protein 2	+	+	+	+	+	+	+	+
*fliR*	Flagellar biosynthetic FliR protein	+	+	+	+	+	+	+	+
*fliT*	Flagellar FliT protein	+	+	+	+	+	+	+	+
*fliZ*	FliZ protein	+	+	+	+	+	+	+	+
*lolA*	Outer membrane lipoprotein carrier protein	+	+	+	+	+	+	+	+
*motB*	Chemotaxis MotA protein	+	+	+	+	+	+	+	+
*sdiA*	LuxR family transcriptional regulator	+	+	+	+	+	+	+	+
*slyB*	Outer membrane lipoprotein SlyB	+	+	+	+	+	+	+	+
*tolC*	Outer membrane channel protein	+	+	+	+	+	+	+	+
*msbA*	Survival in macrophage	+	+	+	+	+	+	−	+
*mviN*	Protective immunity and colonization	+	+	+	+	+	+	+	+
*cpa*	Plasminogen activator	+	+	+	+	−	+	−	−
*hly*	Hemolysin	+	+	+	+	+	+	−	+
*ompA*	Adhesion cell, biofilm formation	+	+	+	+	+	+	+	+
*ompX*	Adhesion cell	+	+	+	+	+	+	+	+
*cheR*	Chemotaxis protein methyltransferase	+	+	−	+	+	−	+	−
*cheY*	Response regulator of chemotaxis family	+	+	+	+	+	+	+	+
*cheB*	Desiccation tolerance	+	+	+	+	+	+	+	+
*lpxA*	Epithelial cell invasion and lipid A production	+	+	+	+	+	+	+	+
*nanA,K,T*	Exogenous sialic acid utilization	+	+	+	+	−	+	−	+
*ibpA*	Small heat shock protein	+	+	+	+	+	+	+	+
*wzzB*	Desiccation tolerance	+	+	+	+	+	+	+	+
*fic*	Cell filamentation protein	+	+	+	+	+	+	+	+
*relB*	RelE antitoxin	+	+	+	+	+	+	−	+

+, presence and −, absence.

In the *Salmonella* strains, 120 virulence genes and eight pathogenicity islands were detected. The virulence genes clustered as adherence, magnesium uptake, resistance to antimicrobial peptides, secretion system, stress protein, toxin, resistance to complement killing. The *shdA* gene associated with persistence of the bacteria in the intestine was only present in the 510535-21 strain. The *gogB*, *pipB*, *ssaCTU*, *ssel/srfH*, *sseL*, *sspH2*, *shdA*, *sopD2*, and *sseK1,2* genes were not found in the 510536-21, 510537-21, 510538-21, 510539-21, and 510540-21 strains associated with the secretion system, effector proteins, adherence, and host survival (Table 6).

**Table 6:** Putative virulence and distribution of other genes in six strains of *S.* Typhimurium by WGS.

	Genes	510535	510536	510537	510538	510539	510540
*Virulence*	*avrAbcfAbcfBbcfCbcfDbcfEbcfFbcfGcsgAcsgBcsgCcsgDcsgEcsgFcsgGfimCfimDfimFfimHfimIgrvAinvAinvBinvCinvEinvFinvGinvHinvIinvJlpfAlpfBlpfClpfDlpfEmgtBmgtCmig-14misLorgAorgBorgCpefApefBpefCpefDpipB2prgHprgIprgJprgKratBrcksicAsicPsifAsifBsinHsipA/sspAsipB/sspBsipC/sspCsipDslrPsodCIsopAsopB/sigDsopDsopE2spaOspaPspaQspaRspaSspiC/ssaBsptPspvBspvCspvRssaDssaEssaGssaHssaIssaJssaKssaLssaMssaNssaOssaPssaQssaRssaSssaVsscAsscBsseAsseBsseCsseDsseEsseFsseGsseJsseLsspH1steAsteBsteC*	+	+	+	+	+	+
	*gogBpipBssaCTUssel/srfHsseK2sseLsspH2*	+	−	+	+	+	+
	*shdA*	+	−	−	+	−	−
	*sopD2*	+	−	−	+	+	+
	*sseK1*	+	−	+	+	−	+
	*TTSS(Type III secretion system)*	+	+	+	+	+	+
*Pathogenicity islands*	*SPI-1*	+	+	+	+	+	+
	*SPI-2*	+	+	+	+	+	+
	*SPI-3*	+	+	+	+	+	+
	*SPI-4*	+	+	−	+	+	−
	*SPI-5*	+	+	+	+	+	+
	*SPI-9*	+	+	+	+	+	+
	*SPI-13*	+	+	+	+	+	+
	*SPI-14*	+	+	+	+	+	+

+, presence and −, absence.

### Detection of Plasmids and Mobile Genetics Elements

The Col(pHHAD28) plasmids and seven MGEs (IS903, IS26, ISEsa2, IS5075, ISEsa1, ISPpu12, and IS102) were detected in only three *C. sakazakii* strains. The IncFIB(pCTU1) plasmid and one MGE (IS481) were detected in the *C. malonaticus* strain.

All the *Salmonella* strains exhibited the IncFII(S) plasmids and five similar MGEs (ISSen7, ISSty2, ISEcI10, MITEEcl, and ISSen1; Table 7).

**Table 7:** Plasmids and mobile genetic elements of *Cronobacter* spp. and *S.* Typhimurium.

Bacteria	ID strain	Plasmid	Plasmids accession number	Mobile genetic elements
*Cronobacter sakazakii*	510197-19	Col(pHDA28)	KU674895	IS903, IS26, ISEsa2, IS5075, ISEsa1, ISPpu12, IS102
	510199-19	Col(pHDA28)	KU674895	IS903, IS26. ISEsa2, IS5075, ISEsa1, ISPpu12, IS102
	510290-18	Col(pHDA28)	KU674895	ISEsa2, IS5075, ISEsa1, ISPpu12, IS102
	510556	----		ISEsa1
*Cronobacter malonaticus*	510557-19	IncFIB(pCTU1)	FN543094	IS481
*Salmonella* Typhimurium	510535-21	IncFII(S)	FN543094	ISSen7, ISSty2,ISEcI10, MITEEcl, ISSen1
	510536-21	IncFIB(S) INCFII(S)	FN432031 CP000858	ISSen7, ISSen1, MITEEc1, ISEcl10
	510537-21	IncFII(S)	FN543094	ISSen7, MITEEcl, ISSen1, ISEcI10, ISSty2
	510538-21	IncFII(S)	FN543094	ISSen7, MITEEcl, ISEcI10, ISSen1
	510539-21	IncFII(S)	FN543094	ISSen7, MITEEcl, ISSen1, ISSty2, ISEcI10
	510540-21	IncFII(S)	FN543094	ISSen7, MITEEcl, ISSen1, ISEcI10

### CRISPR-Cas Loci Profiling

Genome analysis showed CRISPR-Cas systems in all of the genomes. In *Cronobacter* spp., 80% (*n* = 4/5) of the genomes revealed the presence of up to three arrays, which were characterized by the same repeated sequences but at different positions in the genome (Table 8). In the case of *Salmonella* spp. isolates, 100% (*n* = 6/6) of the genomes showed two arrays associated with the CRISPR-Cas systems in different positions but characterized by the same number of repeated sequences and spacers, with up to 28 repeated sequences and 27 spacers.

**Table 8:** CRISPR-Cas systems identified in the *Cronobacter* spp. and *Salmonella* spp. genomes.

Strains	Operon structure type	Position	Maximum number of spacers per strain	Number of CRISPR arrays per strain	Repeat consensus	*cas* genes
510197-*Cronobacter* spp.	Type I-F CAS	77362-76641	12	13	GTTCACTGCCGTACAGGCAGCTTAGAAA	DEDDh*,cas3,cas8e,cse2gr11,cas7,cas5,cas6e,cas1,cas2*
	Type I-E CAS	171199-172847	27	28	CTGTTCCCCGCGCGAGCGGGGATAAACCG	
		199092-200862	29	30	GTGTTCCCCGCGCGAGCGGGGATAAACCG	
510199-*Cronobacter* spp.	Type I-E CAS	482009-482670	11	12	GTTCACTGCCGTACAGGCAGCTTAGAAA	*cas2,cas1,cas6e,cas5,cas7,cse2gr11,cas8e,cas3*,DEDDh
		77461-79109	27	28	CGGTTTATCCCCGCTCGCGCGGGGAA	
		105476-107002	25	26	CGGTTTATCCCCGCTCGCGCGGGGAACAG	
510290-*Cronobacter* spp.	Type I-F CAS	480714-481435	12	13	GTTCACTGCCGTACAGGCAGCTTAGAAA	DEDDh, *csa3,cas3,cas8e,cse2gr11,cas7,DinG,cas6,cas1*
	-Type I-E CAS	173384-175032	27	28	CTGTTCCCCGCGCGAGCGGGGATAAACCG	
		201277-203047	29	30	GTGTTCCCCGCGCGAGCGGGGATAAACCG	
510556-*Cronobacter* spp.	Type I-F CAS	161191-162091	9	10	CTGTTCCCCGCGCGAGCGGGGATAAACCG	*cas2*, *csa3,DinG,cas3,DEDDh,csa3,cas2,cas1,cas6e,cas5,cas7,cse2gr11,cas8e*
	-Type I-E CAS	7728-8277	15	16	GTGTTCCCCGCGCGAGCGGGGATAAACCG	
		199129-200044	15	16	GTTCACTGCCGTACAGGCAGCTTAGAAA	
510557-*Cronobacter* spp.	-Type I-E CAS	6074-7051	17	18	GTGTTCCCCGCGCGAGCGGGGATAAACCG	*cas8e,cas3,cse2gr11,cas7,cas5,cas6e,cas1,cas2,csa3,WYL,csa3,DEDDh*, *DinG*
		165212-166676	25	26	CTGTTCCCCGCGCGAGCGGGGATAAACCG	
510535-*Salmonella* spp.	Type I-E CAS	241410-242857	24	25	GTGTTCCCCGCGCCAGCGGGGATAAACCG	*DinG,c2c9_V-U4,cas3,cas14j,cas3,PD-DExK,cas2,cas1,cas6e,cas5,cas7,cse2gr11,cas8e*
		259017-260604				
			27	28	GTGTTCCCCGCGCCAGCGGGGATAAACCG	
510536-*Salmonella* spp.	Type I-E CAS	7220-8807	27	28	GTGTTCCCCGCGCCAGCGGGGATAAACCG	*c2c9_V-U4,cas14j,csa3 DinG,DEDDh,cas3,cas2,cas1,cas6e,cas5,cas7,cse2gr11,cas8e,PD-DExK*
		27933-29380	24	25	GTGTTCCCCGCGCCAGCGGGGATAAACCG	
510537-*Salmonella* spp.	Type I-E CAS	5320-6907	27	28	GTGTTCCCCGCGCCAGCGGGGATAAACCG	*c2c9_V-U4,cas14j,DinG,c2c9_V-U4,DEDDh,cas3,DEDDh,DinG,cas3,cas8e,cse2gr11,cas7,cas5,cas6e,cas1,cas2,cas14j*
		23067-24514	24	25	GTGTTCCCCGCGCCAGCGGGGATAAACCG	
510538-*Salmonella* spp.	Type I-E CAS	166775-165328	24	25	GTGTTCCCCGCGCCAGCGGGGATAAACCG	*c2c9_V-U4,cas14j,c2c9_V-U4,DinG,DEDDh,cas3,PD-DExK,cas2,cas1,cas6e,cas5,cas7,cse2gr11,cas8e*
		184522-182935	27	28	GTGTTCCCCGCGCCAGCGGGGATAAACCG	
510539-*Salmonella* spp.	Type I-E CAS	5479-7066	24	25	GTGTTCCCCGCGCCAGCGGGGATAAACCG	*c2c9_V-U4,c2c9_V-U4,csa3,DinG,cas14j,cas3,cas8e,DnG,cse2gr11,cas7,cas5,cas6e,cas1,cas2,DEDDh,cas3*
		8963-10410	27	28	GTGTTCCCCGCGCCAGCGGGGATAAACCG	
510540-*Salmonella* spp.	-Type I-E CAS	161008-162455	24	25	GTGTTCCCCGCGCCAGCGGGGATAAACCG	*c2c9_V-U4,c2c9_V-U4,DinG,DEDDh,cas3,DEDDh,cas14j,cas3,PD-DExK,cas2,cas1,cas6e,cas5,cas7,cse2gr11,cas8e,cas3*
		178615-180202	27	28	GTGTTCCCCGCGCCAGCGGGGATAAACCG	

Characteristic repeated sequences of the identified CRISPR arrays and their position in the genome.

**Figure 3:**
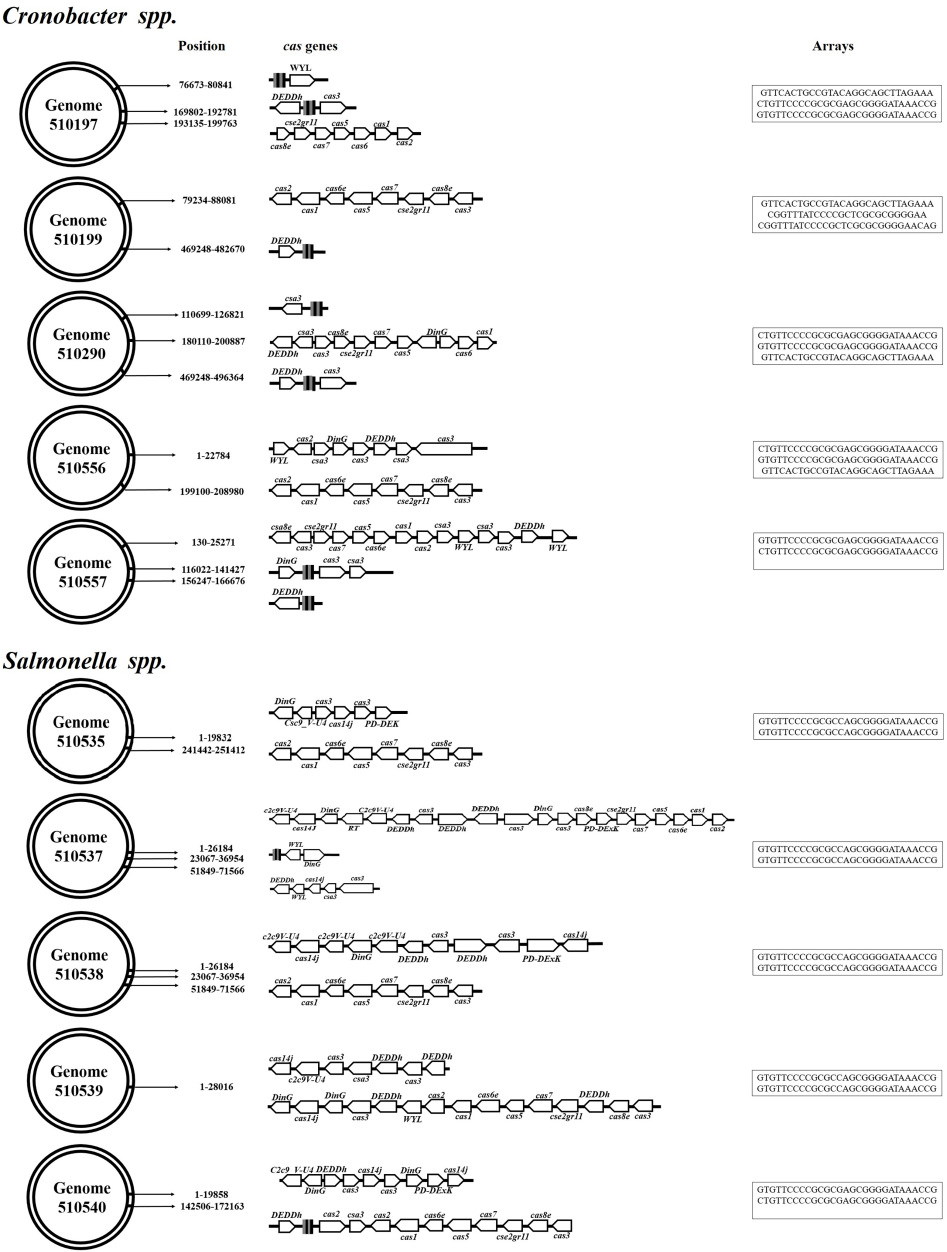
CRISPR-Cas systems identified in *Cronobacter* spp. and *Salmonella* spp. genomes. The identified systems belong to the I-E and I-F CRISPR-Cas systems.

Using the CRISPRmap program, the repeated sequences and associated *cas* genes allowed us to determine that the CRISPR systems identified in *Cronobacter* spp. genomes belonged to type I-E and I-F in which 60% (*n* = 3/5) were characterized by the presence of both types of CRISPR-Cas systems. However, the opposite was observed in the *Salmonella* genomes, which were associated with the presence of type I-E systems. As for the associated *cas* genes, type I-E systems of the *Salmonella* genomes showed a larger number of genes associated with the CRISPR arrays (Figure 3).

The analysis of the spacers with CRISPRminer revealed bacteriophage sequences with bacteriophages that are characteristic of *Salmonella* and *Enterobacteriaceae* in three *Cronobacter* spp. genomes (Supplementary Table 1). However, when searching for the PAM sequences, the spacers of the CRISPR arrays identified in the *Cronobacter* genomes were also characterized by phages associated with the *Klebsiella*, *Streptococcus*, and *Acinetobacter* genera. The spacers of the *Salmonella* spp. genomes were associated with the *crAss* and *Acanthamoeba polyphaga moumouvirus* phages and bacteriophages that are characteristic of *Salmonella* and *E. coli* (Supplementary Table 2). These results are correlated with the phages that were identified in the study genomes (Supplementary Table 3).

## Discussion

WGS enables the molecular typing of bacterial strains on a routine basis for use in epidemiology and real-time infection control. Its usefulness has also been demonstrated in identifying antimicrobial resistance markers, virulence, and the genetic prediction of antibiotic susceptibility test results (Lepuschitz et al., 2020).

In the present study, we identified and characterized *C. sakazakii* ST1 and ST31, *C. malonaticus* ST60, and *S.* Typhimurium ST19 strains from commercially available PIF. The three strains of the pathovar *C. sakazakii* ST1 were isolated from different batches of PIF which came same manufacturer. This may reflect either the widespread occurrence of this sequence type in PIF manufacturing environment or that a common ingredient was contaminated with the same strain (Sonbol et al., 2013). *Cronobacter sakazakii* ST1 has frequently been found in PIF commercialized in different countries, in the PIF processing environment, and in invasive clinical cases such as fatal meningitis and septicemia (Joseph and Forsythe, 2012; Sonbol et al., 2013; Fei et al., 2015; Csorba et al., 2021; Holý et al., 2021; Parra-Flores et al., 2021b). A survey of *Cronobacter* in the Americas showed the majority of reports isolations were from North America (57.4%, *n* = 465) and Brazil (42.6%, *n* = 465). There were a total of 75 sequence types, with the most frequently reported being the *C. sakazakii* pathovars ST4 (CC4) and ST1 (CC1; Costa et al., 2021).

The cgMLST scheme analysis clustered the three *C. sakazakii* ST1 strains closely to the *C. sakazakii* ST1 strains isolated from the food alert in Chile on 2017 with one to three alleles differences (Parra-Flores et al., 2018b). When analyzing the isolated strains of human infections in a European multicenter study using cgMLST, eight *C. sakazakii* ST1 isolates were found among all the *C. sakazakii* strains. Of these eight ST1 isolates, two strains were analyzed from an outbreak affecting two newborns suffering from necrotizing enterocolitis in Austria in 2009, which differed by only one allele. In addition, three ST1 isolates from Austria and one ST1 from Denmark differed by 203 alleles with the ATCC BAA-894 strain, which was isolated from PIF associated with a fatal case of an infant in the United States in 2001. *Cronobacter sakazakii* ST31 has also been isolated from clinical cases with fatal outcomes; however, it has been less prevalent in PIF and the environment (Sonbol et al., 2013; Fei et al., 2015; Ogrodzki and Forsythe, 2015; Lepuschitz et al., 2019). In this context, *C. sakazakii* ST1, ST8, and especially ST4 are the STs with the highest risk of causing disease in infants (Joseph and Forsythe, 2011; Forsythe, 2018; Lachowska et al., 2021). *Cronobacter malonaticus* ST60 has also been isolated in powdered milk, food, and the environment, but with less significant clinical cases than *C. malonaticus* ST7 and *C. sakazakii* ST1 (Forsythe, 2018; Costa et al., 2021).

*Salmonella enterica* is the most frequently identified cause of food poisoning in the European Union; serotype Typhimurium ST19 is most often associated with disease and death (Carroll et al., 2017; de Frutos et al., 2018) and commonly identified in human clinical samples, animals, food, and the environment (Panzenhagen et al., 2018; Monte et al., 2020). The cgMLST scheme analysis revealed a cluster of six *S*. Typhimurium ST19 with one to two allele differences. In this context, the present study is the first to identify *S*. Typhimurium ST19 in PIF, whereas recent reports of *Salmonella* outbreaks in PIF have involved *S*. Agona (Brouard et al., 2007; Jourdan-da Silva et al., 2018). We also presumptively identified two *Salmonella* monophasic variant strains. Monophasic *Salmonella* has been identified in different human cases in the United States, Spain, Brazil, and Thailand and characterization of these strains revealed resistance to multiple antibiotics (Mossong et al., 2007).

All five *C. sakazakii* strains analyzed in the present study were resistant to cephalothin and only two strains to ampicillin. The resistance of *C. sakazakii* to cephalothin and ampicillin has been reported in several studies, also suggesting an almost intrinsic resistance of the *Cronobacter* spp. genus to cephalothin (Kim et al., 2008; Molloy et al., 2009; Flores et al., 2011; Chon et al., 2012). Parra-Flores et al. (2020) found that 100% of the *C. sakazakii* strains isolated in powdered infant formula distributed in Latin America were resistant to cefotaxime and ampicillin, 60% to cefepime, 40% to amikacin, and 20% to cephalothin. Furthermore, one of the strains was resistant to six of the 12 evaluated antibiotics and another strain was resistant to five antibiotics. Multiple drug resistance (MDR) is a cause for concern; in a case of neonatal meningitis caused by *C. sakazakii* in China, the isolated strains were resistant to eight antibiotics (Zeng et al., 2018).

For the antibiotic resistance genes, all *C. sakazakii* strains and the *C. malonaticus* strain exhibited the same efflux, antibiotic inactivation, and antibiotic target alteration genes that confer antibiotic resistance to β-lactams, fluoroquinolones, aminoglycosides, imidazoles, and disinfectants such as triclosan. The *marA* gene, whose transcription function regulates multidrug efflux, modulates membrane permeability and activates the transcription of the AcrAB-TolC efflux pump that plays an important role in antibiotic resistance (Wang X. et al., 2021). Several authors have detected the *msbA*, *emrR*, *H-NS*, *emrB*, *marA*, *CRP*, *PBP3*, *H-NS*, and *msrB* genes that are associated with antibiotic resistance by using efflux pumps, regulatory systems, and antibiotic target protection genes (Aly et al., 2019; Lepuschitz et al., 2019; Parra-Flores et al., 2021b). All strains exhibited the *glpT* gene that encodes fosfomycin resistance. This is relevant because fosfomycin is considered effective in patients with MDR bacterial infections (Falagas et al., 2019). We also identified the *mcr-9.1* gene that confers resistance to colistin and the *bla_CSA_* and *bla_CMA_* genes that confer resistance to cephalothin. The *mcr-9.1* gene can generate resistance to colistin in several enteropathogens and can silently circulate undetected unless induced by colistin (Carroll et al., 2019; Kieffer et al., 2019). The presence of mobile genes resistant to colistin (*mcr*) is a worldwide public concern because colistin is considered as a last resort to treat infections caused by multidrug-resistant *Enterobacteriaceae* (Borowiak et al., 2020). The *bla_CSA_* and *bla_CMA_* genes were first described in 2014 and are associated with β-lactamase class C resistance; they are not inducible and are regarded as cephalosporinases (Müller et al., 2014). Jang et al. (2020) found variants of class C *bla* resistance genes identified as *CSA-2* or *CSA-1* and *CMA* in all the analyzed strains. Holý et al. (2021) encountered *bla_CSA_* genes in all the *C. sakazakii* strains isolated in powdered milk produced in the Czech Republic between 2010 and 2014.

In the present study, all six *S*. Typhimurium ST19 strains were resistant to oxacillin, five to ampicillin, four to cephalothin, and one to gentamicin. *S*. Typhimurium ST19 has shown extensive resistance to a variety of critically important antimicrobials (Monte et al., 2020). Jain et al. (2018) reported that all the evaluated *S*. Typhimurium ST19 strains were resistant to 7 of the 15 tested antibiotics and encountered only strains that were susceptible to ampicillin and gentamicin; this contrasts with our study in which strains were resistant to these two antibiotics.

The *Salmonella* strains carried many genes such as *aac(6′)-Iaa* that encodes resistance to aminoglycosides (e.g., gentamicin), which have been found in multidrug-resistant *S.* Typhimurium strains and caused a foodborne outbreak at a banquet in China in 2017 (Xiang et al., 2020). Wei et al. (2019) reported the presence of β-*lactamase ampC* genes the same as in our study; in addition to finding β-*lactamase ampC-1*, we found that it co-harbored with β-*lactamase ampH*. The β-*lactamase ampC* is chromosomally encoded in several gram-negative bacteria, including *Enterobacter* spp., *Citrobacter freundii*, or *Serratia marcescens*. Furthermore, plasmid-encoded *ampC* genes can be horizontally transferred to other *Enterobacteriaceae* without the presence of chromosomally encoded *ampC*, such as *Klebsiella* and *Salmonella*, resulting in a highly effective and dynamic dissemination mechanism (Ingram et al., 2011). We also found the *uhpT* and *glpT* genes that encode fosfomycin resistance. The UhpT and GlpT transporters facilitate fosfomycin incorporation into bacterial cells (Silver, 2017). The reduced expression or introduction of *glpT* or *uhpT* mutations and the efflux pump can decrease fosfomycin uptake and thus lower antibiotic susceptibility (Garallah and Al-Jubori, 2020). Several studies have now shown that the overuse of antibiotics in the food chain and the presence of several antibiotic resistance operons promote multiresistance to these drugs (Ferri et al., 2017).

Among the 30 virulence genes detected in *C. sakazakii*, we identified *C. sakazakii ompA* and *ompX*, which encode proteins for basolateral adherence in cell lines and a possible involvement in the blood–brain barrier penetration (Mange et al., 2006; Kim et al., 2010). The Cpa protein is related to *C. sakazakii* serum resistance and invasion. Recent studies have suggested that the *cpa* locus could be considered specific for *C. sakazakii* and *C. universalis* but is absent in *C. malonaticus* (Franco et al., 2011). Jang et al. (2020) noted that highly virulent *C. sakazakii* ST8 clinical strains that carry the pESA3 plasmid do not possess the *cpa* gene; this indicates that the disease can be associated with other virulence factors. Hemolysins (Hly) are outer membrane proteins occurring in various pathogens belonging to the *Enterobacteriaceae* family that have hemolytic ability, such as *E. coli*, *Klebsiella*, and some gram-positive pathogens (Mare et al., 2020; Mazzantini et al., 2020). Other important genes of the *C. sakazakii* strains isolated from PIF are *nanAKT* that encode for exogenous sialic acid utilization. Sialic acid is naturally present in breast milk and supplemented in PIF because of its association with brain development (Forsythe, 2018). Sialic acid also regulates the expression of enzymes, such as sialidase and adhesins, or inhibits transcription factors of the *fimB* gene that mediates epithelial cell adherence and invasion. *Escherichia coli* K1 use this compound to modify their cell surface (Severi et al., 2007; Sohanpal et al., 2007).

In our study, we found the *fic* toxin encoding gene and the *relB* gene that encodes for the *relE* antitoxin. The *fic* gene encodes for the toxin component of the toxin-antitoxin bicistronic operon. The toxin-antitoxin (TA) systems are small genetic elements found in plasmids, phage genomes, and in the chromosomes of different bacterial species. The TA genes play a fundamental role in the physiology of bacterial stress response, such as in stabilizing horizontally acquired mobile genetic elements and participating in a persistence phenotype in some species, including *E. coli* and *Salmonella* (Deter et al., 2017; Walling and Butler, 2019). Finkelstein et al. (2019) noted in preliminary studies with *C. sakazakii* isolates that 2 typical toxin genes, *fic* and *hipA*, followed the evolutionary lines of the species and that *C. sakazakii* ST1 strains were the only strains containing the 22 TA homologs.

In the *Salmonella* strains, 110 of the 121 detected virulence genes were similar in all the strains, including the *invA*, *ssaAR ssrAB*, *sipAC*, *sopBE*, *spvBC*, and *rck* genes. Genes such as *invA*, *sipA*, *sopB*, and *sopE* are associated with adherence in epithelial cells and phagocyte invasion. *Salmonella* pathogenicity islands (SPI) are virulence gene clusters acquired by horizontal transfer that promote virulence in *Salmonella* spp. The SPI-2 gene encodes genes such as *ssaR* and *ssrA* that promote survival and replication within the macrophages and their dissemination in the host (Barilli et al., 2018). The *spvC* gene enables the survival and rapid growth of *Salmonella* in the host (Hu et al., 2021), while the *spvB* gene is known as an intracellular toxin that encodes an enzyme that ADP-ribosylates actin and destabilizes the cytoskeleton of eukaryotic cells (Lesnick et al., 2001).

The *rck* gene is defined as an invasin that generates a colonization niche or as a cyclomodulin with genotoxic activity (Mambu et al., 2020). To date, 24 SPIs have been identified in *Salmonella* of which SPI-1 and SPI-2 are the most important for virulence traits. SPI-1 is involved in the entire *Salmonella* infection process, including invasion, macrophage proliferation, and host responses. Both SPI-1 and SPI-2 encode different secretion systems, such as T3SS, that transport effector proteins to the host cells (Cheng et al., 2019; Lou et al., 2019).

Only the *C. sakazakii* ST1 strains carried the COL(pHHAD28) plasmids, which are associated with antibiotic resistance genes; these have previously been detected in *C. sakazakii* strains isolated from dairy products in Chile (Ramsamy et al., 2020; Khezri et al., 2021; Parra-Flores et al., 2021b). The *C. malonaticus* strain harbored a plasmid homologous to pESA3 called IncFIB (pCTU1), which encodes iron acquisition virulence genes necessary for pathogen survival, but not an external protease known as plasminogen activator (*cpa*) that enhances pathogen propagation in the host (Franco et al., 2011). Meanwhile, all the *Salmonella* strains showed IncFII(S) plasmid incompatibility, which is associated with several antibiotic resistance genes, such as *aac(6′)-Iaa* and *mcr-9*, and β-lactamase, such as *ampC* (Cha et al., 2020; Richter et al., 2020).

Bacterial CRISPR-Cas systems are considered as mechanisms of acquired immunity because they provide them with the ability to avoid bacteriophage infection and the acquisition of mobile genetic material from plasmids. This immunity is due to the information stored in the matrices of these systems, specifically in the spacer sequences. It was possible to determine that repeated sequences and *cas* genes in *Cronobacter* spp. were associated with two types of systems previously identified as I-E and I-F (Ogrodzki and Forsythe, 2016; Zeng et al., 2017), including cases in which the same strain showed both systems. The opposite was observed in *Salmonella* spp. genomes that were characterized only by the presence of type I-E (Louwen et al., 2014). Although both genera carry systems of the same type, there are differences between the associated *cas* gene operons because the *Salmonella* spp. systems show a larger number of genes. However, these systems are characterized by the presence of the *cas1* and *cas2* genes, which are involved in integrating and processing the information that is integrated into functional CRISPR arrays. *Salmonella enterica* and *E. coli* are closely related and harbor the IE-type CRISPR-Cas system in a similar manner (Makarova et al., 2015). The array sizes of the *Cronobacter* spp. genomes are smaller. However, when showing more than two arrays, they tend to have a larger number of spacer, suggesting that they have been exposed to a larger number of invasive elements than *S.* Typhimurium. Although it was not possible to determine that all the spacers of the systems are similar to bacteriophage sequences, there are cases in which they are specific to these genera. Of particular interest, some of the *S.* Typhimurium spacer were associated with *CrAssphages* and other phages associated with the human intestinal microbiota (Guerin et al., 2018). It is known that *Salmonella* is recognized as an intestinal pathogen; therefore, this is an ecosystem where bacteria can acquire external genetic material and integrate it into their genome by various horizontal transfer mechanisms and by other means such as CRISPR-Cas systems (Louwen et al., 2014). The *cas1* and *cas2* genes are indispensable for integrating and processing the information acquired by the bacteria; in their absence, the system loses the ability to acquire information (Parra-Flores et al., 2021b). The search for prophages in the studied genomes showed that they may have few intact phages. *Cronobacter* spp. tend to show a larger number of intact and incomplete prophages, which are not only characteristic of this genus but also of *Salmonella*. Therefore, our data suggest that these genomes carry the necessary information to prevent these bacteriophages from infecting both *Cronobacter* spp. and *Salmonella* strains. In addition, this information can be useful in the future when using gene therapy as a therapeutic option for infections caused by these pathogens that are difficult to treat (Gordillo Altamirano and Barr, 2019).

In the present study, WGS allowed us to determine multiple virulence and antibiotic resistance genes in bacterial pathogens isolated from PIF intended for consumption by infants aged less than 6 months and distributed throughout Latin America. In our opinion, the identification of *C. sakazakii* and *S.* Typhimurium in PIFs not only violates current health regulations but also endanger the health of infants consuming these products. It is therefore necessary that health authorities conduct more preventive control activities related to these products and carry out campaigns that emphasize the use of rehydration water at 70°C for infant formula. This recommendation by the World Health Organization highlights that the 70°C temperature has a proven effect in significantly decreasing the risk of disease by pathogens in reconstituted PIF (FAO/WHO, 2004, 2006). Control of *Salmonella* and *Cronobacter* during the production of milk powder and PIF is through microbiological sampling according to Codex Alimentarius Commission (2007) of finished product, as well as ingredients and intermediate products (International Standards for Organization (ISO), 2017; Podolak and Black, 2017). The use of zoning production facilities to focus environmental sampling according to risk is used (Cordier, 2008; FDA, 2019).

## Conclusion

*C. sakazakii*, *C. malonaticus*, and *S.* Typhimurium strains isolated from PIF exhibit antibiotic resistance and various virulence genes and resistance to β-lactam antibiotics. Continuous monitoring of these powdered infant formulas is necessary due to the risk associated with pathogen contamination of the product and consumption by the immunologically vulnerable child population.

## Data Availability Statement

The datasets presented in this study can be found in online repositories. The names of the repository/repositories and accession number(s) can be found in the article/Supplementary Material.

## Author Contributions

JP-F, OH, SA, SL, AC-C, JM-R, AC, and SF conceived the experiments and prepared the manuscript. JP-F, AC-F, PC-S, AC-C, AP, SL, and WR conducted the laboratory work. JP-F, OH, SA, AC-C, JX-C, JM-R, AC, WR, and SF drafted the manuscript. All authors contributed to the article and approved the submitted version.

## Funding

This research was funded by the Research Directorate of the Universidad del Bío-Bío, Projects 191520 4/R and GI 195420/EF.

## Conflict of Interest

The authors declare that the research was conducted in the absence of any commercial or financial relationships that could be construed as a potential conflict of interest.

## Publisher’s Note

All claims expressed in this article are solely those of the authors and do not necessarily represent those of their affiliated organizations, or those of the publisher, the editors and the reviewers. Any product that may be evaluated in this article, or claim that may be made by its manufacturer, is not guaranteed or endorsed by the publisher.

## Footnotes

^1^ https://pubmlst.org/organisms/cronobacter-spp/

^2^ http://enterobase.warwick.ac.uk/species/index/senterica

^3^ http://pubmlst.org/cronobacter/

^4^ http://www.genomicepidemiology.org
