# Supplementary material for: Genomic Characterization of Cronobacter spp. and Salmonella spp. Strains Isolated From Powdered Infant Formula in Chile
Source: Front Microbiol. 2022 Jun 2;13:884721. doi: 10.3389/fmicb.2022.884721 (PMC9201451; doi:10.3389/fmicb.2022.884721)
Supplement: Supplementary file 1 [file Table_1.DOCX]

**Supplementary Table 1.** Spacer sequences that comprise the CRISPR arrays. These spacers are associated with sequences corresponding to phages.

| **510197-*Cronobacter spp.*** | | | | | | | |
| --- | --- | --- | --- | --- | --- | --- | --- |
| **Phage ID** | **Phage Name** | **Spacer ID** | **Identity** | **Coverage** | **Hit Length** | **Hit Postition** | **Spacer Sequence** |
| ref\|NC_005886.2\| | Burkholderia phage BcepB1A | 1.2\|126764\|57\|510290-18_contig1 | 100 | 0.56 | 21 | 26036-26016 | CGCGGCTTACCGGCAAGCTTCACACATTAGAAAACCCCGCTTCGGCGGGGTTTTTGC |
| ref\|NC_006949.1\| | Enterobacteria phage ES18 | 1.1\|126643\|67\|510290-18_contig1 | 95.8 | 0.48 | 24 | 13259-13282 | AGAACCCGGCTTACCGGTCAGCTTCACACTTTAGAAAACCCCGCTCCGGCGGGGTTTTTGCTTTTTG |
| ref\|NC_013693.1\| | Shigella phage Ag3 | 1.2\|126764\|57\|510290-18_contig1 | 100 | 0.56 | 25 | 102546-102522 | CGCGGCTTACCGGCAAGCTTCACACATTAGAAAACCCCGCTTCGGCGGGGTTTTTGC |
| ref\|NC_015296.1\| | Salmonella phage ViI | 1.2\|126764\|57\|510290-18_contig1 | 100 | 0.56 | 23 | 119969-119947 | CGCGGCTTACCGGCAAGCTTCACACATTAGAAAACCCCGCTTCGGCGGGGTTTTTGC |
| ref\|NC_016073.1\| | Salmonella phage SFP10 | 1.2\|126764\|57\|510290-18_contig1 | 100 | 0.56 | 24 | 100120-100097 | CGCGGCTTACCGGCAAGCTTCACACATTAGAAAACCCCGCTTCGGCGGGGTTTTTGC |
| ref\|NC_016073.1\| | Salmonella phage SFP10 | 1.2\|126764\|57\|510290-18_contig1 | 96 | 0.56 | 25 | 119251-119227 | CGCGGCTTACCGGCAAGCTTCACACATTAGAAAACCCCGCTTCGGCGGGGTTTTTGC |
| ref\|NC_016570.1\| | Escherichia phage Cba120 | 1.2\|126764\|57\|510290-18_contig1 | 100 | 0.56 | 25 | 118905-118881 | CGCGGCTTACCGGCAAGCTTCACACATTAGAAAACCCCGCTTCGGCGGGGTTTTTGC |
| ref\|NC_016570.1\| | Escherichia phage Cba120 | 1.2\|126764\|57\|510290-18_contig1 | 100 | 0.56 | 24 | 99779-99756 | CGCGGCTTACCGGCAAGCTTCACACATTAGAAAACCCCGCTTCGGCGGGGTTTTTGC |
| ref\|NC_016767.1\| | Erwinia phage PEp14 | 1.1\|126643\|67\|510290-18_contig1 | 96 | 0.48 | 25 | 41823-41847 | AGAACCCGGCTTACCGGTCAGCTTCACACTTTAGAAAACCCCGCTCCGGCGGGGTTTTTGCTTTTTG |
| ref\|NC_019452.1\| | Escherichia phage PhaxI | 1.2\|126764\|57\|510290-18_contig1 | 100 | 0.56 | 23 | 106314-106292 | CGCGGCTTACCGGCAAGCTTCACACATTAGAAAACCCCGCTTCGGCGGGGTTTTTGC |
| ref\|NC_019522.1\| | Pectobacterium phage ZF40 | 1.1\|126643\|67\|510290-18_contig1 | 93.6 | 0.48 | 31 | 16744-16774 | AGAACCCGGCTTACCGGTCAGCTTCACACTTTAGAAAACCCCGCTCCGGCGGGGTTTTTGCTTTTTG |
| ref\|NC_019530.1\| | Salmonella phage PhiSH19 | 1.2\|126764\|57\|510290-18_contig1 | 100 | 0.56 | 24 | 101569-101546 | CGCGGCTTACCGGCAAGCTTCACACATTAGAAAACCCCGCTTCGGCGGGGTTTTTGC |
| ref\|NC_019545.1\| | Salmonella phage SPN3UB | 1.1\|126643\|67\|510290-18_contig1 | 90.3 | 0.48 | 31 | 16060-16089 | AGAACCCGGCTTACCGGTCAGCTTCACACTTTAGAAAACCCCGCTCCGGCGGGGTTTTTGCTTTTTG |
| ref\|NC_019704.1\| | Enterobacteria phage mEp237 | 1.1\|126643\|67\|510290-18_contig1 | 95.8 | 0.48 | 24 | 20066-20089 | AGAACCCGGCTTACCGGTCAGCTTCACACTTTAGAAAACCCCGCTCCGGCGGGGTTTTTGCTTTTTG |
| ref\|NC_019706.1\| | Enterobacteria phage mEp043 c-1 | 1.1\|126643\|67\|510290-18_contig1 | 95.8 | 0.48 | 24 | 20227-20250 | AGAACCCGGCTTACCGGTCAGCTTCACACTTTAGAAAACCCCGCTCCGGCGGGGTTTTTGCTTTTTG |
| ref\|NC_019710.1\| | Enterobacteria phage HK140 | 1.1\|126643\|67\|510290-18_contig1 | 95.8 | 0.48 | 24 | 18617-18640 | AGAACCCGGCTTACCGGTCAGCTTCACACTTTAGAAAACCCCGCTCCGGCGGGGTTTTTGCTTTTTG |
| ref\|NC_019717.1\| | Enterobacteria phage HK225 | 1.1\|126643\|67\|510290-18_contig1 | 95.8 | 0.48 | 24 | 20072-20095 | AGAACCCGGCTTACCGGTCAGCTTCACACTTTAGAAAACCCCGCTCCGGCGGGGTTTTTGCTTTTTG |
| ref\|NC_019720.1\| | Enterobacterial phage mEp213 | 1.1\|126643\|67\|510290-18_contig1 | 95.8 | 0.48 | 24 | 20227-20250 | AGAACCCGGCTTACCGGTCAGCTTCACACTTTAGAAAACCCCGCTCCGGCGGGGTTTTTGCTTTTTG |
| ref\|NC_019910.1\| | Salmonella phage SKML-39 | 1.2\|126764\|57\|510290-18_contig1 | 100 | 0.56 | 24 | 25500-25523 | CGCGGCTTACCGGCAAGCTTCACACATTAGAAAACCCCGCTTCGGCGGGGTTTTTGC |
| ref\|NC_019910.1\| | Salmonella phage SKML-39 | 1.2\|126764\|57\|510290-18_contig1 | 100 | 0.56 | 24 | 25912-25935 | CGCGGCTTACCGGCAAGCTTCACACATTAGAAAACCCCGCTTCGGCGGGGTTTTTGC |
| ref\|NC_019925.1\| | Dickeya virus Limestone | 1.2\|126764\|57\|510290-18_contig1 | 100 | 0.56 | 24 | 96986-96963 | CGCGGCTTACCGGCAAGCTTCACACATTAGAAAACCCCGCTTCGGCGGGGTTTTTGC |
| ref\|NC_019927.1\| | Cronobacter phage ENT47670 | 5.15\|202404\|32\|510290-18_contig5 | 96.9 | 1 | 32 | 1081-1112 | ATTACAACCAGATCCCCGGCACTACGCAGACA |
| ref\|NC_020083.1\| | Serratia phage phiMAM1 | 1.1\|126643\|67\|510290-18_contig1 | 100 | 0.48 | 25 | 61400-61424 | AGAACCCGGCTTACCGGTCAGCTTCACACTTTAGAAAACCCCGCTCCGGCGGGGTTTTTGCTTTTTG |
| ref\|NC_020083.1\| | Serratia phage phiMAM1 | 1.2\|126764\|57\|510290-18_contig1 | 100 | 0.56 | 21 | 104385-104405 | CGCGGCTTACCGGCAAGCTTCACACATTAGAAAACCCCGCTTCGGCGGGGTTTTTGC |
| ref\|NC_021190.1\| | Enterobacteria phage phi80 | 1.1\|126643\|67\|510290-18_contig1 | 95.8 | 0.48 | 24 | 21550-21573 | AGAACCCGGCTTACCGGTCAGCTTCACACTTTAGAAAACCCCGCTCCGGCGGGGTTTTTGCTTTTTG |
| ref\|NC_022343.1\| | Klebsiella phage 0507-KN2-1 | 1.2\|126764\|57\|510290-18_contig1 | 100 | 0.56 | 24 | 119272-119295 | CGCGGCTTACCGGCAAGCTTCACACATTAGAAAACCCCGCTTCGGCGGGGTTTTTGC |
| ref\|NC_022768.1\| | Salmonella phage Maynard | 1.2\|126764\|57\|510290-18_contig1 | 100 | 0.56 | 25 | 38331-38355 | CGCGGCTTACCGGCAAGCTTCACACATTAGAAAACCCCGCTTCGGCGGGGTTTTTGC |
| ref\|NC_023589.1\| | Shigella phage pSb-1 | 1.2\|126764\|57\|510290-18_contig1 | 100 | 0.56 | 21 | 13717-13697 | CGCGGCTTACCGGCAAGCTTCACACATTAGAAAACCCCGCTTCGGCGGGGTTTTTGC |
| ref\|NC_023601.1\| | Pseudomonas phage phiPsa374 | 1.2\|126764\|57\|510290-18_contig1 | 100 | 0.56 | 23 | 4106-4084 | CGCGGCTTACCGGCAAGCTTCACACATTAGAAAACCCCGCTTCGGCGGGGTTTTTGC |
| ref\|NC_023856.1\| | Salmonella phage vB_SalM_SJ2 | 1.2\|126764\|57\|510290-18_contig1 | 100 | 0.56 | 23 | 115289-115311 | CGCGGCTTACCGGCAAGCTTCACACATTAGAAAACCCCGCTTCGGCGGGGTTTTTGC |
| ref\|NC_023856.1\| | Salmonella phage vB_SalM_SJ2 | 1.2\|126764\|57\|510290-18_contig1 | 100 | 0.56 | 23 | 128053-128075 | CGCGGCTTACCGGCAAGCTTCACACATTAGAAAACCCCGCTTCGGCGGGGTTTTTGC |
| ref\|NC_024122.1\| | Salmonella phage vB_SalM_SJ3 | 1.2\|126764\|57\|510290-18_contig1 | 96 | 0.56 | 25 | 101158-101182 | CGCGGCTTACCGGCAAGCTTCACACATTAGAAAACCCCGCTTCGGCGGGGTTTTTGC |
| ref\|NC_025452.1\| | Dickeya phage RC-2014 | 1.2\|126764\|57\|510290-18_contig1 | 100 | 0.56 | 23 | 72114-72092 | CGCGGCTTACCGGCAAGCTTCACACATTAGAAAACCCCGCTTCGGCGGGGTTTTTGC |
| ref\|NC_027119.1\| | Salmonella phage Det7 | 1.2\|126764\|57\|510290-18_contig1 | 100 | 0.56 | 24 | 12710-12733 | CGCGGCTTACCGGCAAGCTTCACACATTAGAAAACCCCGCTTCGGCGGGGTTTTTGC |
| ref\|NC_027119.1\| | Salmonella phage Det7 | 1.2\|126764\|57\|510290-18_contig1 | 100 | 0.56 | 23 | 5528-5550 | CGCGGCTTACCGGCAAGCTTCACACATTAGAAAACCCCGCTTCGGCGGGGTTTTTGC |
| ref\|NC_027995.1\| | Escherichia phage vB_EcoM_ECO1230-10 | 1.2\|126764\|57\|510290-18_contig1 | 100 | 0.56 | 25 | 915-891 | CGCGGCTTACCGGCAAGCTTCACACATTAGAAAACCCCGCTTCGGCGGGGTTTTTGC |
| ref\|NC_029042.1\| | Salmonella phage 38 | 1.2\|126764\|57\|510290-18_contig1 | 100 | 0.56 | 24 | 56118-56141 | CGCGGCTTACCGGCAAGCTTCACACATTAGAAAACCCCGCTTCGGCGGGGTTTTTGC |
| ref\|NC_031007.1\| | Erwinia phage vB_EamM_EarlPhillipIV | 1.2\|126764\|57\|510290-18_contig1 | 100 | 0.56 | 21 | 103081-103101 | CGCGGCTTACCGGCAAGCTTCACACATTAGAAAACCCCGCTTCGGCGGGGTTTTTGC |
| ref\|NC_031026.1\| | Salmonella phage phSE-2 | 1.2\|126764\|57\|510290-18_contig1 | 100 | 0.56 | 21 | 29587-29567 | CGCGGCTTACCGGCAAGCTTCACACATTAGAAAACCCCGCTTCGGCGGGGTTTTTGC |
| ref\|NC_031045.1\| | Salmonella phage GG32 | 1.2\|126764\|57\|510290-18_contig1 | 100 | 0.56 | 24 | 138788-138765 | CGCGGCTTACCGGCAAGCTTCACACATTAGAAAACCCCGCTTCGGCGGGGTTTTTGC |
| ref\|NC_031045.1\| | Salmonella phage GG32 | 1.2\|126764\|57\|510290-18_contig1 | 96 | 0.56 | 25 | 925-901 | CGCGGCTTACCGGCAAGCTTCACACATTAGAAAACCCCGCTTCGGCGGGGTTTTTGC |
| ref\|NC_031048.1\| | Enterobacter phage Arya | 1.2\|126764\|57\|510290-18_contig1 | 100 | 0.56 | 25 | 25914-25938 | CGCGGCTTACCGGCAAGCTTCACACATTAGAAAACCCCGCTTCGGCGGGGTTTTTGC |
| ref\|NC_031053.1\| | Klebsiella phage PKP126 | 1.2\|126764\|57\|510290-18_contig1 | 100 | 0.56 | 25 | 34418-34442 | CGCGGCTTACCGGCAAGCTTCACACATTAGAAAACCCCGCTTCGGCGGGGTTTTTGC |
| ref\|NC_031128.1\| | Salmonella phage vB_SalM_PM10 | 1.2\|126764\|57\|510290-18_contig1 | 100 | 0.56 | 23 | 65572-65594 | CGCGGCTTACCGGCAAGCTTCACACATTAGAAAACCCCGCTTCGGCGGGGTTTTTGC |
| ref\|NC_031264.1\| | Brucella phage BiPBO1 | 1.2\|126764\|57\|510290-18_contig1 | 100 | 0.56 | 21 | 34753-34733 | CGCGGCTTACCGGCAAGCTTCACACATTAGAAAACCCCGCTTCGGCGGGGTTTTTGC |
| ref\|NC_031924.1\| | Salmonella phage IME207 | 1.2\|126764\|57\|510290-18_contig1 | 96.2 | 0.56 | 26 | 36253-36278 | CGCGGCTTACCGGCAAGCTTCACACATTAGAAAACCCCGCTTCGGCGGGGTTTTTGC |
| ref\|NC_031940.1\| | Salmonella phage 118970_sal3 | 1.1\|126643\|67\|510290-18_contig1 | 93.3 | 0.48 | 30 | 73966-73995 | AGAACCCGGCTTACCGGTCAGCTTCACACTTTAGAAAACCCCGCTCCGGCGGGGTTTTTGCTTTTTG |
| ref\|NC_031940.1\| | Salmonella phage 118970_sal3 | 1.2\|126764\|57\|510290-18_contig1 | 96.2 | 0.56 | 26 | 73966-73991 | CGCGGCTTACCGGCAAGCTTCACACATTAGAAAACCCCGCTTCGGCGGGGTTTTTGC |
| ref\|NC_031940.1\| | Salmonella phage 118970_sal3 | 1.2\|126764\|57\|510290-18_contig1 | 95.8 | 0.56 | 24 | 34503-34526 | CGCGGCTTACCGGCAAGCTTCACACATTAGAAAACCCCGCTTCGGCGGGGTTTTTGC |
| **510290-*Cronobacter spp.*** | | | | | | | |
| ref\|NC_005886.2\| | Burkholderia phage BcepB1A | 1.2\|126764\|57\|510290-18_contig1 | 100.0 | 0.561403508772 | 21 | 26036-26016 | CGCGGCTTACCGGCAAGCTTCACACATTAGAAAACCCCGCTTCGGCGGGGTTTTTGC |
| ref\|NC_006949.1\| | Enterobacteria phage ES18 | 1.1\|126643\|67\|510290-18_contig1 | 95.83 | 0.477611940299 | 24 | 13259-13282 | AGAACCCGGCTTACCGGTCAGCTTCACACTTTAGAAAACCCCGCTCCGGCGGGGTTTTTGCTTTTTG |
| ref\|NC_013693.1\| | Shigella phage Ag3 | 1.2\|126764\|57\|510290-18_contig1 | 100.0 | 0.561403508772 | 25 | 102546-102522 | CGCGGCTTACCGGCAAGCTTCACACATTAGAAAACCCCGCTTCGGCGGGGTTTTTGC |
| ref\|NC_015296.1\| | Salmonella phage ViI | 1.2\|126764\|57\|510290-18_contig1 | 100.0 | 0.561403508772 | 23 | 119969-119947 | CGCGGCTTACCGGCAAGCTTCACACATTAGAAAACCCCGCTTCGGCGGGGTTTTTGC |
| ref\|NC_016073.1\| | Salmonella phage SFP10 | 1.2\|126764\|57\|510290-18_contig1 | 100.0 | 0.561403508772 | 24 | 100120-100097 | CGCGGCTTACCGGCAAGCTTCACACATTAGAAAACCCCGCTTCGGCGGGGTTTTTGC |
| ref\|NC_016073.1\| | Salmonella phage SFP10 | 1.2\|126764\|57\|510290-18_contig1 | 96.0 | 0.561403508772 | 25 | 119251-119227 | CGCGGCTTACCGGCAAGCTTCACACATTAGAAAACCCCGCTTCGGCGGGGTTTTTGC |
| ref\|NC_016570.1\| | Escherichia phage Cba120 | 1.2\|126764\|57\|510290-18_contig1 | 100.0 | 0.561403508772 | 25 | 118905-118881 | CGCGGCTTACCGGCAAGCTTCACACATTAGAAAACCCCGCTTCGGCGGGGTTTTTGC |
| ref\|NC_016570.1\| | Escherichia phage Cba120 | 1.2\|126764\|57\|510290-18_contig1 | 100.0 | 0.561403508772 | 24 | 99779-99756 | CGCGGCTTACCGGCAAGCTTCACACATTAGAAAACCCCGCTTCGGCGGGGTTTTTGC |
| ref\|NC_016767.1\| | Erwinia phage PEp14 | 1.1\|126643\|67\|510290-18_contig1 | 96.0 | 0.477611940299 | 25 | 41823-41847 | AGAACCCGGCTTACCGGTCAGCTTCACACTTTAGAAAACCCCGCTCCGGCGGGGTTTTTGCTTTTTG |
| ref\|NC_019452.1\| | Escherichia phage PhaxI | 1.2\|126764\|57\|510290-18_contig1 | 100.0 | 0.561403508772 | 23 | 106314-106292 | CGCGGCTTACCGGCAAGCTTCACACATTAGAAAACCCCGCTTCGGCGGGGTTTTTGC |
| ref\|NC_019522.1\| | Pectobacterium phage ZF40 | 1.1\|126643\|67\|510290-18_contig1 | 93.55 | 0.477611940299 | 31 | 16744-16774 | AGAACCCGGCTTACCGGTCAGCTTCACACTTTAGAAAACCCCGCTCCGGCGGGGTTTTTGCTTTTTG |
| ref\|NC_019530.1\| | Salmonella phage PhiSH19 | 1.2\|126764\|57\|510290-18_contig1 | 100.0 | 0.561403508772 | 24 | 101569-101546 | CGCGGCTTACCGGCAAGCTTCACACATTAGAAAACCCCGCTTCGGCGGGGTTTTTGC |
| ref\|NC_019545.1\| | Salmonella phage SPN3UB | 1.1\|126643\|67\|510290-18_contig1 | 90.32 | 0.477611940299 | 31 | 16060-16089 | AGAACCCGGCTTACCGGTCAGCTTCACACTTTAGAAAACCCCGCTCCGGCGGGGTTTTTGCTTTTTG |
| ref\|NC_019704.1\| | Enterobacteria phage mEp237 | 1.1\|126643\|67\|510290-18_contig1 | 95.83 | 0.477611940299 | 24 | 20066-20089 | AGAACCCGGCTTACCGGTCAGCTTCACACTTTAGAAAACCCCGCTCCGGCGGGGTTTTTGCTTTTTG |
| ref\|NC_019706.1\| | Enterobacteria phage mEp043 c-1 | 1.1\|126643\|67\|510290-18_contig1 | 95.83 | 0.477611940299 | 24 | 20227-20250 | AGAACCCGGCTTACCGGTCAGCTTCACACTTTAGAAAACCCCGCTCCGGCGGGGTTTTTGCTTTTTG |
| ref\|NC_019710.1\| | Enterobacteria phage HK140 | 1.1\|126643\|67\|510290-18_contig1 | 95.83 | 0.477611940299 | 24 | 18617-18640 | AGAACCCGGCTTACCGGTCAGCTTCACACTTTAGAAAACCCCGCTCCGGCGGGGTTTTTGCTTTTTG |
| ref\|NC_019717.1\| | Enterobacteria phage HK225 | 1.1\|126643\|67\|510290-18_contig1 | 95.83 | 0.477611940299 | 24 | 20072-20095 | AGAACCCGGCTTACCGGTCAGCTTCACACTTTAGAAAACCCCGCTCCGGCGGGGTTTTTGCTTTTTG |
| ref\|NC_019720.1\| | Enterobacterial phage mEp213 | 1.1\|126643\|67\|510290-18_contig1 | 95.83 | 0.477611940299 | 24 | 20227-20250 | AGAACCCGGCTTACCGGTCAGCTTCACACTTTAGAAAACCCCGCTCCGGCGGGGTTTTTGCTTTTTG |
| ref\|NC_019910.1\| | Salmonella phage SKML-39 | 1.2\|126764\|57\|510290-18_contig1 | 100.0 | 0.561403508772 | 24 | 25500-25523 | CGCGGCTTACCGGCAAGCTTCACACATTAGAAAACCCCGCTTCGGCGGGGTTTTTGC |
| ref\|NC_019910.1\| | Salmonella phage SKML-39 | 1.2\|126764\|57\|510290-18_contig1 | 100.0 | 0.561403508772 | 24 | 25912-25935 | CGCGGCTTACCGGCAAGCTTCACACATTAGAAAACCCCGCTTCGGCGGGGTTTTTGC |
| ref\|NC_019925.1\| | Dickeya virus Limestone | 1.2\|126764\|57\|510290-18_contig1 | 100.0 | 0.561403508772 | 24 | 96986-96963 | CGCGGCTTACCGGCAAGCTTCACACATTAGAAAACCCCGCTTCGGCGGGGTTTTTGC |
| ref\|NC_019927.1\| | Cronobacter phage ENT47670 | 5.15\|202404\|32\|510290-18_contig5 | 96.88 | 1.0 | 32 | 1081-1112 | ATTACAACCAGATCCCCGGCACTACGCAGACA |
| ref\|NC_020083.1\| | Serratia phage phiMAM1 | 1.1\|126643\|67\|510290-18_contig1 | 100.0 | 0.477611940299 | 25 | 61400-61424 | AGAACCCGGCTTACCGGTCAGCTTCACACTTTAGAAAACCCCGCTCCGGCGGGGTTTTTGCTTTTTG |
| ref\|NC_020083.1\| | Serratia phage phiMAM1 | 1.2\|126764\|57\|510290-18_contig1 | 100.0 | 0.561403508772 | 21 | 104385-104405 | CGCGGCTTACCGGCAAGCTTCACACATTAGAAAACCCCGCTTCGGCGGGGTTTTTGC |
| ref\|NC_021190.1\| | Enterobacteria phage phi80 | 1.1\|126643\|67\|510290-18_contig1 | 95.83 | 0.477611940299 | 24 | 21550-21573 | AGAACCCGGCTTACCGGTCAGCTTCACACTTTAGAAAACCCCGCTCCGGCGGGGTTTTTGCTTTTTG |
| ref\|NC_022343.1\| | Klebsiella phage 0507-KN2-1 | 1.2\|126764\|57\|510290-18_contig1 | 100.0 | 0.561403508772 | 24 | 119272-119295 | CGCGGCTTACCGGCAAGCTTCACACATTAGAAAACCCCGCTTCGGCGGGGTTTTTGC |
| ref\|NC_022768.1\| | Salmonella phage Maynard | 1.2\|126764\|57\|510290-18_contig1 | 100.0 | 0.561403508772 | 25 | 38331-38355 | CGCGGCTTACCGGCAAGCTTCACACATTAGAAAACCCCGCTTCGGCGGGGTTTTTGC |
| ref\|NC_023589.1\| | Shigella phage pSb-1 | 1.2\|126764\|57\|510290-18_contig1 | 100.0 | 0.561403508772 | 21 | 13717-13697 | CGCGGCTTACCGGCAAGCTTCACACATTAGAAAACCCCGCTTCGGCGGGGTTTTTGC |
| ref\|NC_023601.1\| | Pseudomonas phage phiPsa374 | 1.2\|126764\|57\|510290-18_contig1 | 100.0 | 0.561403508772 | 23 | 4106-4084 | CGCGGCTTACCGGCAAGCTTCACACATTAGAAAACCCCGCTTCGGCGGGGTTTTTGC |
| ref\|NC_023856.1\| | Salmonella phage vB_SalM_SJ2 | 1.2\|126764\|57\|510290-18_contig1 | 100.0 | 0.561403508772 | 23 | 115289-115311 | CGCGGCTTACCGGCAAGCTTCACACATTAGAAAACCCCGCTTCGGCGGGGTTTTTGC |
| ref\|NC_023856.1\| | Salmonella phage vB_SalM_SJ2 | 1.2\|126764\|57\|510290-18_contig1 | 100.0 | 0.561403508772 | 23 | 128053-128075 | CGCGGCTTACCGGCAAGCTTCACACATTAGAAAACCCCGCTTCGGCGGGGTTTTTGC |
| ref\|NC_024122.1\| | Salmonella phage vB_SalM_SJ3 | 1.2\|126764\|57\|510290-18_contig1 | 96.0 | 0.561403508772 | 25 | 101158-101182 | CGCGGCTTACCGGCAAGCTTCACACATTAGAAAACCCCGCTTCGGCGGGGTTTTTGC |
| ref\|NC_025452.1\| | Dickeya phage RC-2014 | 1.2\|126764\|57\|510290-18_contig1 | 100.0 | 0.561403508772 | 23 | 72114-72092 | CGCGGCTTACCGGCAAGCTTCACACATTAGAAAACCCCGCTTCGGCGGGGTTTTTGC |
| ref\|NC_027119.1\| | Salmonella phage Det7 | 1.2\|126764\|57\|510290-18_contig1 | 100.0 | 0.561403508772 | 24 | 12710-12733 | CGCGGCTTACCGGCAAGCTTCACACATTAGAAAACCCCGCTTCGGCGGGGTTTTTGC |
| ref\|NC_027119.1\| | Salmonella phage Det7 | 1.2\|126764\|57\|510290-18_contig1 | 100.0 | 0.561403508772 | 23 | 5528-5550 | CGCGGCTTACCGGCAAGCTTCACACATTAGAAAACCCCGCTTCGGCGGGGTTTTTGC |
| ref\|NC_027995.1\| | Escherichia phage vB_EcoM_ECO1230-10 | 1.2\|126764\|57\|510290-18_contig1 | 100.0 | 0.561403508772 | 25 | 915-891 | CGCGGCTTACCGGCAAGCTTCACACATTAGAAAACCCCGCTTCGGCGGGGTTTTTGC |
| ref\|NC_029042.1\| | Salmonella phage 38 | 1.2\|126764\|57\|510290-18_contig1 | 100.0 | 0.561403508772 | 24 | 56118-56141 | CGCGGCTTACCGGCAAGCTTCACACATTAGAAAACCCCGCTTCGGCGGGGTTTTTGC |
| ref\|NC_031007.1\| | Erwinia phage vB_EamM_EarlPhillipIV | 1.2\|126764\|57\|510290-18_contig1 | 100.0 | 0.561403508772 | 21 | 103081-103101 | CGCGGCTTACCGGCAAGCTTCACACATTAGAAAACCCCGCTTCGGCGGGGTTTTTGC |
| ref\|NC_031026.1\| | Salmonella phage phSE-2 | 1.2\|126764\|57\|510290-18_contig1 | 100.0 | 0.561403508772 | 21 | 29587-29567 | CGCGGCTTACCGGCAAGCTTCACACATTAGAAAACCCCGCTTCGGCGGGGTTTTTGC |
| ref\|NC_031045.1\| | Salmonella phage GG32 | 1.2\|126764\|57\|510290-18_contig1 | 100.0 | 0.561403508772 | 24 | 138788-138765 | CGCGGCTTACCGGCAAGCTTCACACATTAGAAAACCCCGCTTCGGCGGGGTTTTTGC |
| ref\|NC_031045.1\| | Salmonella phage GG32 | 1.2\|126764\|57\|510290-18_contig1 | 96.0 | 0.561403508772 | 25 | 925-901 | CGCGGCTTACCGGCAAGCTTCACACATTAGAAAACCCCGCTTCGGCGGGGTTTTTGC |
| ref\|NC_031048.1\| | Enterobacter phage Arya | 1.2\|126764\|57\|510290-18_contig1 | 100.0 | 0.561403508772 | 25 | 25914-25938 | CGCGGCTTACCGGCAAGCTTCACACATTAGAAAACCCCGCTTCGGCGGGGTTTTTGC |
| ref\|NC_031053.1\| | Klebsiella phage PKP126 | 1.2\|126764\|57\|510290-18_contig1 | 100.0 | 0.561403508772 | 25 | 34418-34442 | CGCGGCTTACCGGCAAGCTTCACACATTAGAAAACCCCGCTTCGGCGGGGTTTTTGC |
| ref\|NC_031128.1\| | Salmonella phage vB_SalM_PM10 | 1.2\|126764\|57\|510290-18_contig1 | 100.0 | 0.561403508772 | 23 | 65572-65594 | CGCGGCTTACCGGCAAGCTTCACACATTAGAAAACCCCGCTTCGGCGGGGTTTTTGC |
| ref\|NC_031264.1\| | Brucella phage BiPBO1 | 1.2\|126764\|57\|510290-18_contig1 | 100.0 | 0.561403508772 | 21 | 34753-34733 | CGCGGCTTACCGGCAAGCTTCACACATTAGAAAACCCCGCTTCGGCGGGGTTTTTGC |
| ref\|NC_031924.1\| | Salmonella phage IME207 | 1.2\|126764\|57\|510290-18_contig1 | 96.15 | 0.561403508772 | 26 | 36253-36278 | CGCGGCTTACCGGCAAGCTTCACACATTAGAAAACCCCGCTTCGGCGGGGTTTTTGC |
| ref\|NC_031940.1\| | Salmonella phage 118970_sal3 | 1.1\|126643\|67\|510290-18_contig1 | 93.33 | 0.477611940299 | 30 | 73966-73995 | AGAACCCGGCTTACCGGTCAGCTTCACACTTTAGAAAACCCCGCTCCGGCGGGGTTTTTGCTTTTTG |
| ref\|NC_031940.1\| | Salmonella phage 118970_sal3 | 1.2\|126764\|57\|510290-18_contig1 | 96.15 | 0.561403508772 | 26 | 73966-73991 | CGCGGCTTACCGGCAAGCTTCACACATTAGAAAACCCCGCTTCGGCGGGGTTTTTGC |
| ref\|NC_031940.1\| | Salmonella phage 118970_sal3 | 1.2\|126764\|57\|510290-18_contig1 | 95.83 | 0.561403508772 | 24 | 34503-34526 | CGCGGCTTACCGGCAAGCTTCACACATTAGAAAACCCCGCTTCGGCGGGGTTTTTGC |
| **510199-*Cronobacter spp.*** | | | | | | | |
| ref\|NC_005886.2\| | Burkholderia phage BcepB1A | 1.2\|128058\|57\|510199-19_contig1 | 100.0 | 0.561403508772 | 21 | 26036-26016 | CGCGGCTTACCGGCAAGCTTCACACATTAGAAAACCCCGCTTCGGCGGGGTTTTTGC |
| ref\|NC_006949.1\| | Enterobacteria phage ES18 | 1.1\|127937\|67\|510199-19_contig1 | 95.83 | 0.477611940299 | 24 | 13259-13282 | AGAACCCGGCTTACCGGTCAGCTTCACACTTTAGAAAACCCCGCTCCGGCGGGGTTTTTGCTTTTTG |
| ref\|NC_013693.1\| | Shigella phage Ag3 | 1.2\|128058\|57\|510199-19_contig1 | 100.0 | 0.561403508772 | 25 | 102546-102522 | CGCGGCTTACCGGCAAGCTTCACACATTAGAAAACCCCGCTTCGGCGGGGTTTTTGC |
| ref\|NC_015296.1\| | Salmonella phage ViI | 1.2\|128058\|57\|510199-19_contig1 | 100.0 | 0.561403508772 | 23 | 119969-119947 | CGCGGCTTACCGGCAAGCTTCACACATTAGAAAACCCCGCTTCGGCGGGGTTTTTGC |
| ref\|NC_016073.1\| | Salmonella phage SFP10 | 1.2\|128058\|57\|510199-19_contig1 | 100.0 | 0.561403508772 | 24 | 100120-100097 | CGCGGCTTACCGGCAAGCTTCACACATTAGAAAACCCCGCTTCGGCGGGGTTTTTGC |
| ref\|NC_016073.1\| | Salmonella phage SFP10 | 1.2\|128058\|57\|510199-19_contig1 | 96.0 | 0.561403508772 | 25 | 119251-119227 | CGCGGCTTACCGGCAAGCTTCACACATTAGAAAACCCCGCTTCGGCGGGGTTTTTGC |
| ref\|NC_016570.1\| | Escherichia phage Cba120 | 1.2\|128058\|57\|510199-19_contig1 | 100.0 | 0.561403508772 | 25 | 118905-118881 | CGCGGCTTACCGGCAAGCTTCACACATTAGAAAACCCCGCTTCGGCGGGGTTTTTGC |
| ref\|NC_016570.1\| | Escherichia phage Cba120 | 1.2\|128058\|57\|510199-19_contig1 | 100.0 | 0.561403508772 | 24 | 99779-99756 | CGCGGCTTACCGGCAAGCTTCACACATTAGAAAACCCCGCTTCGGCGGGGTTTTTGC |
| ref\|NC_016767.1\| | Erwinia phage PEp14 | 1.1\|127937\|67\|510199-19_contig1 | 96.0 | 0.477611940299 | 25 | 41823-41847 | AGAACCCGGCTTACCGGTCAGCTTCACACTTTAGAAAACCCCGCTCCGGCGGGGTTTTTGCTTTTTG |
| ref\|NC_019452.1\| | Escherichia phage PhaxI | 1.2\|128058\|57\|510199-19_contig1 | 100.0 | 0.561403508772 | 23 | 106314-106292 | CGCGGCTTACCGGCAAGCTTCACACATTAGAAAACCCCGCTTCGGCGGGGTTTTTGC |
| ref\|NC_019522.1\| | Pectobacterium phage ZF40 | 1.1\|127937\|67\|510199-19_contig1 | 93.55 | 0.477611940299 | 31 | 16744-16774 | AGAACCCGGCTTACCGGTCAGCTTCACACTTTAGAAAACCCCGCTCCGGCGGGGTTTTTGCTTTTTG |
| ref\|NC_019530.1\| | Salmonella phage PhiSH19 | 1.2\|128058\|57\|510199-19_contig1 | 100.0 | 0.561403508772 | 24 | 101569-101546 | CGCGGCTTACCGGCAAGCTTCACACATTAGAAAACCCCGCTTCGGCGGGGTTTTTGC |
| ref\|NC_019545.1\| | Salmonella phage SPN3UB | 1.1\|127937\|67\|510199-19_contig1 | 90.32 | 0.477611940299 | 31 | 16060-16089 | AGAACCCGGCTTACCGGTCAGCTTCACACTTTAGAAAACCCCGCTCCGGCGGGGTTTTTGCTTTTTG |
| ref\|NC_019704.1\| | Enterobacteria phage mEp237 | 1.1\|127937\|67\|510199-19_contig1 | 95.83 | 0.477611940299 | 24 | 20066-20089 | AGAACCCGGCTTACCGGTCAGCTTCACACTTTAGAAAACCCCGCTCCGGCGGGGTTTTTGCTTTTTG |
| ref\|NC_019706.1\| | Enterobacteria phage mEp043 c-1 | 1.1\|127937\|67\|510199-19_contig1 | 95.83 | 0.477611940299 | 24 | 20227-20250 | AGAACCCGGCTTACCGGTCAGCTTCACACTTTAGAAAACCCCGCTCCGGCGGGGTTTTTGCTTTTTG |
| ref\|NC_019710.1\| | Enterobacteria phage HK140 | 1.1\|127937\|67\|510199-19_contig1 | 95.83 | 0.477611940299 | 24 | 18617-18640 | AGAACCCGGCTTACCGGTCAGCTTCACACTTTAGAAAACCCCGCTCCGGCGGGGTTTTTGCTTTTTG |
| ref\|NC_019717.1\| | Enterobacteria phage HK225 | 1.1\|127937\|67\|510199-19_contig1 | 95.83 | 0.477611940299 | 24 | 20072-20095 | AGAACCCGGCTTACCGGTCAGCTTCACACTTTAGAAAACCCCGCTCCGGCGGGGTTTTTGCTTTTTG |
| ref\|NC_019720.1\| | Enterobacterial phage mEp213 | 1.1\|127937\|67\|510199-19_contig1 | 95.83 | 0.477611940299 | 24 | 20227-20250 | AGAACCCGGCTTACCGGTCAGCTTCACACTTTAGAAAACCCCGCTCCGGCGGGGTTTTTGCTTTTTG |
| ref\|NC_019910.1\| | Salmonella phage SKML-39 | 1.2\|128058\|57\|510199-19_contig1 | 100.0 | 0.561403508772 | 24 | 25500-25523 | CGCGGCTTACCGGCAAGCTTCACACATTAGAAAACCCCGCTTCGGCGGGGTTTTTGC |
| ref\|NC_019910.1\| | Salmonella phage SKML-39 | 1.2\|128058\|57\|510199-19_contig1 | 100.0 | 0.561403508772 | 24 | 25912-25935 | CGCGGCTTACCGGCAAGCTTCACACATTAGAAAACCCCGCTTCGGCGGGGTTTTTGC |
| ref\|NC_019925.1\| | Dickeya virus Limestone | 1.2\|128058\|57\|510199-19_contig1 | 100.0 | 0.561403508772 | 24 | 96986-96963 | CGCGGCTTACCGGCAAGCTTCACACATTAGAAAACCCCGCTTCGGCGGGGTTTTTGC |
| ref\|NC_019927.1\| | Cronobacter phage ENT47670 | 3.9\|77976\|35\|510199-19_contig7 | 96.88 | 0.914285714286 | 32 | 1112-1081 | CACTGTCTGCGTAGTGCCGGGGATCTGGTTGTAAT |
| ref\|NC_020083.1\| | Serratia phage phiMAM1 | 1.1\|127937\|67\|510199-19_contig1 | 100.0 | 0.477611940299 | 25 | 61400-61424 | AGAACCCGGCTTACCGGTCAGCTTCACACTTTAGAAAACCCCGCTCCGGCGGGGTTTTTGCTTTTTG |
| ref\|NC_020083.1\| | Serratia phage phiMAM1 | 1.2\|128058\|57\|510199-19_contig1 | 100.0 | 0.561403508772 | 21 | 104385-104405 | CGCGGCTTACCGGCAAGCTTCACACATTAGAAAACCCCGCTTCGGCGGGGTTTTTGC |
| ref\|NC_021190.1\| | Enterobacteria phage phi80 | 1.1\|127937\|67\|510199-19_contig1 | 95.83 | 0.477611940299 | 24 | 21550-21573 | AGAACCCGGCTTACCGGTCAGCTTCACACTTTAGAAAACCCCGCTCCGGCGGGGTTTTTGCTTTTTG |
| ref\|NC_022343.1\| | Klebsiella phage 0507-KN2-1 | 1.2\|128058\|57\|510199-19_contig1 | 100.0 | 0.561403508772 | 24 | 119272-119295 | CGCGGCTTACCGGCAAGCTTCACACATTAGAAAACCCCGCTTCGGCGGGGTTTTTGC |
| ref\|NC_022768.1\| | Salmonella phage Maynard | 1.2\|128058\|57\|510199-19_contig1 | 100.0 | 0.561403508772 | 25 | 38331-38355 | CGCGGCTTACCGGCAAGCTTCACACATTAGAAAACCCCGCTTCGGCGGGGTTTTTGC |
| ref\|NC_023589.1\| | Shigella phage pSb-1 | 1.2\|128058\|57\|510199-19_contig1 | 100.0 | 0.561403508772 | 21 | 13717-13697 | CGCGGCTTACCGGCAAGCTTCACACATTAGAAAACCCCGCTTCGGCGGGGTTTTTGC |
| ref\|NC_023601.1\| | Pseudomonas phage phiPsa374 | 1.2\|128058\|57\|510199-19_contig1 | 100.0 | 0.561403508772 | 23 | 4106-4084 | CGCGGCTTACCGGCAAGCTTCACACATTAGAAAACCCCGCTTCGGCGGGGTTTTTGC |
| ref\|NC_023856.1\| | Salmonella phage vB_SalM_SJ2 | 1.2\|128058\|57\|510199-19_contig1 | 100.0 | 0.561403508772 | 23 | 115289-115311 | CGCGGCTTACCGGCAAGCTTCACACATTAGAAAACCCCGCTTCGGCGGGGTTTTTGC |
| ref\|NC_023856.1\| | Salmonella phage vB_SalM_SJ2 | 1.2\|128058\|57\|510199-19_contig1 | 100.0 | 0.561403508772 | 23 | 128053-128075 | CGCGGCTTACCGGCAAGCTTCACACATTAGAAAACCCCGCTTCGGCGGGGTTTTTGC |
| ref\|NC_024122.1\| | Salmonella phage vB_SalM_SJ3 | 1.2\|128058\|57\|510199-19_contig1 | 96.0 | 0.561403508772 | 25 | 101158-101182 | CGCGGCTTACCGGCAAGCTTCACACATTAGAAAACCCCGCTTCGGCGGGGTTTTTGC |
| ref\|NC_025452.1\| | Dickeya phage RC-2014 | 1.2\|128058\|57\|510199-19_contig1 | 100.0 | 0.561403508772 | 23 | 72114-72092 | CGCGGCTTACCGGCAAGCTTCACACATTAGAAAACCCCGCTTCGGCGGGGTTTTTGC |
| ref\|NC_027119.1\| | Salmonella phage Det7 | 1.2\|128058\|57\|510199-19_contig1 | 100.0 | 0.561403508772 | 24 | 12710-12733 | CGCGGCTTACCGGCAAGCTTCACACATTAGAAAACCCCGCTTCGGCGGGGTTTTTGC |
| ref\|NC_027119.1\| | Salmonella phage Det7 | 1.2\|128058\|57\|510199-19_contig1 | 100.0 | 0.561403508772 | 23 | 5528-5550 | CGCGGCTTACCGGCAAGCTTCACACATTAGAAAACCCCGCTTCGGCGGGGTTTTTGC |
| ref\|NC_027995.1\| | Escherichia phage vB_EcoM_ECO1230-10 | 1.2\|128058\|57\|510199-19_contig1 | 100.0 | 0.561403508772 | 25 | 915-891 | CGCGGCTTACCGGCAAGCTTCACACATTAGAAAACCCCGCTTCGGCGGGGTTTTTGC |
| ref\|NC_029042.1\| | Salmonella phage 38 | 1.2\|128058\|57\|510199-19_contig1 | 100.0 | 0.561403508772 | 24 | 56118-56141 | CGCGGCTTACCGGCAAGCTTCACACATTAGAAAACCCCGCTTCGGCGGGGTTTTTGC |
| ref\|NC_031007.1\| | Erwinia phage vB_EamM_EarlPhillipIV | 1.2\|128058\|57\|510199-19_contig1 | 100.0 | 0.561403508772 | 21 | 103081-103101 | CGCGGCTTACCGGCAAGCTTCACACATTAGAAAACCCCGCTTCGGCGGGGTTTTTGC |
| ref\|NC_031026.1\| | Salmonella phage phSE-2 | 1.2\|128058\|57\|510199-19_contig1 | 100.0 | 0.561403508772 | 21 | 29587-29567 | CGCGGCTTACCGGCAAGCTTCACACATTAGAAAACCCCGCTTCGGCGGGGTTTTTGC |
| ref\|NC_031045.1\| | Salmonella phage GG32 | 1.2\|128058\|57\|510199-19_contig1 | 100.0 | 0.561403508772 | 24 | 138788-138765 | CGCGGCTTACCGGCAAGCTTCACACATTAGAAAACCCCGCTTCGGCGGGGTTTTTGC |
| ref\|NC_031045.1\| | Salmonella phage GG32 | 1.2\|128058\|57\|510199-19_contig1 | 96.0 | 0.561403508772 | 25 | 925-901 | CGCGGCTTACCGGCAAGCTTCACACATTAGAAAACCCCGCTTCGGCGGGGTTTTTGC |
| ref\|NC_031048.1\| | Enterobacter phage Arya | 1.2\|128058\|57\|510199-19_contig1 | 100.0 | 0.561403508772 | 25 | 25914-25938 | CGCGGCTTACCGGCAAGCTTCACACATTAGAAAACCCCGCTTCGGCGGGGTTTTTGC |
| ref\|NC_031053.1\| | Klebsiella phage PKP126 | 1.2\|128058\|57\|510199-19_contig1 | 100.0 | 0.561403508772 | 25 | 34418-34442 | CGCGGCTTACCGGCAAGCTTCACACATTAGAAAACCCCGCTTCGGCGGGGTTTTTGC |
| ref\|NC_031128.1\| | Salmonella phage vB_SalM_PM10 | 1.2\|128058\|57\|510199-19_contig1 | 100.0 | 0.561403508772 | 23 | 65572-65594 | CGCGGCTTACCGGCAAGCTTCACACATTAGAAAACCCCGCTTCGGCGGGGTTTTTGC |
| ref\|NC_031264.1\| | Brucella phage BiPBO1 | 1.2\|128058\|57\|510199-19_contig1 | 100.0 | 0.561403508772 | 21 | 34753-34733 | CGCGGCTTACCGGCAAGCTTCACACATTAGAAAACCCCGCTTCGGCGGGGTTTTTGC |
| ref\|NC_031924.1\| | Salmonella phage IME207 | 1.2\|128058\|57\|510199-19_contig1 | 96.15 | 0.561403508772 | 26 | 36253-36278 | CGCGGCTTACCGGCAAGCTTCACACATTAGAAAACCCCGCTTCGGCGGGGTTTTTGC |
| ref\|NC_031940.1\| | Salmonella phage 118970_sal3 | 1.1\|127937\|67\|510199-19_contig1 | 93.33 | 0.477611940299 | 30 | 73966-73995 | AGAACCCGGCTTACCGGTCAGCTTCACACTTTAGAAAACCCCGCTCCGGCGGGGTTTTTGCTTTTTG |
| ref\|NC_031940.1\| | Salmonella phage 118970_sal3 | 1.2\|128058\|57\|510199-19_contig1 | 96.15 | 0.561403508772 | 26 | 73966-73991 | CGCGGCTTACCGGCAAGCTTCACACATTAGAAAACCCCGCTTCGGCGGGGTTTTTGC |
| ref\|NC_031940.1\| | Salmonella phage 118970_sal3 | 1.2\|128058\|57\|510199-19_contig1 | 95.83 | 0.561403508772 | 24 | 34503-34526 | CGCGGCTTACCGGCAAGCTTCACACATTAGAAAACCCCGCTTCGGCGGGGTTTTTGC |
