# Supplementary material for: Genomic Characterization of Cronobacter spp. and Salmonella spp. Strains Isolated From Powdered Infant Formula in Chile
Source: Front Microbiol. 2022 Jun 2;13:884721. doi: 10.3389/fmicb.2022.884721 (PMC9201451; doi:10.3389/fmicb.2022.884721)
Supplement: Supplementary file 2 [file Table_2.DOCX]

**Supplementary Table 2.** Protospacer adjacent motifs (PAMs) associated with spacer sequences. The PAM sequences are shown in red at the beginning and end of the sequence.

| **No. De espaciador** | **Secuencia (3´-5´)** | **Posición** | **Phage associated** |
| --- | --- | --- | --- |
| **510197-*Cronobacter spp.*** |  |  |  |
| 10 | TTCGGCGCCCATTCGGCTGACTTCCGTTCAGGCTACGGCTTGACTACC | 171777-171808 | *Klebsiella phage ST11-VIM1phi8.1* |
| 15 | TCAGACGTGCGTGCGAAGGCAAGAGCGGCGCGCTCACGTCCCGAACAG | 172082-172113 | *Cronobacter phage ENT47670* |
| 27 | GGCGCTTCTTGAGTACTTCGCGTTGTCGAAGCCGAGACACTTATTGTT | 172815-172846 | *Cronobacter phage ENT47670* |
| **510199- *Cronobacter spp.*** |  |  |  |
| 2 | CTATTTCAAAATACCAAGCACGACATATAGATACGCAAACCCAACAAC | 79048-79017 |  |
| 8 | GATGATAGCTTCTACACATACTGCGTGAGCTTTACTACCTTCACGTTT | 77828-77797 | *Salmonella phage FSL SP-101* |
| 22 | ACCAAGTCTATAATTGCCGCTGCTGCAAGCGCCGCACCACGAAGTAGA | 77828-77797 | *Salmonella phage FSL SP-101* |
| 29 | CTCACTACGCCACTACATAGAGAGGAGCTATCTACTAATTCATCAGGC | 77400-77369 | *Cronobacter phage ENT47670* |
| **510290- *Cronobacter spp.*** |  |  |  |
| 2 | CTATTTCAAAATACCAAGCACGACATATAGATACGCAAACCCAACAAC | 201367-201398 | *Streptococcus phage Javan115* |
| 8 | GATGATAGCTTCTACACATACTGCGTGAGCTTTACTACCTTCACGTTT | 201733-201764 | *Acinetobacter phage vB_AbaM_Acibel004* |
| 22 | ACCAAGTCTATAATTGCCGCTGCTGCAAGCGCCGCACCACGAAGTAGA | 202587-202618 | *Salmonella phage FSL SP-101* |
| **510556- *Cronobacter spp.*** |  |  |  |
| 14 | GTAAATATGGTAGTGGTTGTCGAGGTTTTAGAAACCCCGATTATCGTA | 199222-199191 |  |
| **510557- *Cronobacter spp.*** |  |  |  |
| 16 | TCTTCAACACGCGCTTGACCGCGCATATCGCGACCGTAGCGACCTTCG | 166156-166187 | *Salmonella typhimurium bacteriophage ES18* |
| 22 | CGGCGTACCGATAACTGCCCGACTTTCTCCATGTCCTTCGGCAGTACG | 166521-166552 | *Enterobacteria phage HK225, complete genome* |
| 24 | TTTTTTTGCTTTTTTGGCTTTCCAAAAAATTTTTACCAAAAAAAATTC | 166643-166674 | *Aureococcus anophagefferens virus isolate BtV-01* |
| **510535-*Salmonella spp.*** |  |  |  |
| 6 | CATATATAAACGCACAAATAAATAGACACGGTAATATAAGCAATAGAA | 242522-242491 | *CrAssphage cr272* |
| 10 | TCTACAGCGGTAAAGTAGTCCGTGATGGCCGTGACCGACCCGCACTGC | 242278-242247 | *Salmonella phage MG40* |
| 12 | CAGACTGAGGTGTAAACCGACGCAACCGGGGTAACCAGGTGACTTTGT | 24156-242125 | *Escherichia phage vB_EcoP_Kapi1* |
| 17 | ATCTATTTAGACAAAAGTTGTAACGTCGTTTTAGTGTCCTTCTAGCTC | 241808-2441777 | *Phage Gifsy-1* |
| **510536- *Salmonella spp.*** |  |  |  |
| 24 | AAATCCGTTTCTAATAAGTGTTTTTAATTAATTATACTATGTTAGATA | 7374-7343 | *Acanthamoeba polyphaga moumouvirus* |
| **510537- *Salmonella spp.*** |  |  |  |
| 24 | AAATCCGTTTCTAATAAGTGTTTTTAATTAATTATACTATGTTAGATA | 673-6784 | *Acanthamoeba polyphaga moumouvirus* |
| **510538- *Salmonella spp.*** |  |  |  |
| 6 | CATATATAAACGCACAAATAAATAGACACGGTAATATAAGCAATAGAA | 166440-166409 | *CrAssphage cr272_1* |
| 10 | TCTACAGCGGTAAAGTAGTCCGTGATGGCCGTGACCGACCCGCACTGC | 166196-166165 | *Salmonella phage MG40* |
| 12 | CAGACTACGGTGCAAGCCGCTACAACCGGGGTAGCCAGGCGACTTCGT | 166074-166043 | *Salmonella phage SW-37* |
| **510539- *Salmonella spp.*** |  |  |  |
| 24 | AAATCCGTTTCTAATAAGTGTTTTTAATTAATTATACTATGTTAGATA | 6912-6943 | *Acanthamoeba polyphaga moumouvirus* |
| **510540- *Salmonella spp.*** |  |  |  |
| 6 | CATATATAAACGCACAAATAAATAGACACGGTAATATAAGCAATAGAA | 162120-162089 | *CrAssphage cr273_1* |
