# Supplementary material for: Genomic Characterization of Cronobacter spp. and Salmonella spp. Strains Isolated From Powdered Infant Formula in Chile
Source: Front Microbiol. 2022 Jun 2;13:884721. doi: 10.3389/fmicb.2022.884721 (PMC9201451; doi:10.3389/fmicb.2022.884721)
Supplement: Supplementary file 3 [file Table_3.DOCX]

**Supplementary Table 3.** Sequences corresponding to phages present in *Cronobacter* and *Salmonella* genomes.

| **Region Length** | **Completeness** | **Score** | **# Total Proteins** | **Region Position** | **Most Common Phage** | **GC %** |
| --- | --- | --- | --- | --- | --- | --- |
| **510197-*Cronobacter spp.*** | | | | | | |
| 19.9Kb | incomplete | 20 | 8 | 604049-624028 | PHAGE_Cronob_phiES15_NC_018454(3) | 46.66% |
| 24.7Kb | intact | 150 | 35 | 615573-640367 | PHAGE_Salmon_118970_sal3_NC_031940(11) | 51.75% |
| 7.2Kb | incomplete | 20 | 9 | 518490-525753 | PHAGE_Erwini_vB_EhrS_49_NC_048197(5) | 51.93% |
| 10.2Kb | incomplete | 60 | 13 | 162105-172348 | PHAGE_Acinet_vB_AbaM_ME3_NC_041884(3) | 49.76% |
| 29.4Kb | incomplete | 30 | 10 | 314140-343626 | PHAGE_Salmon_118970_sal3_NC_031940(2) | 53.66% |
| 12.9Kb | incomplete | 30 | 9 | 169-13149 | PHAGE_Cronob_ENT47670_NC_019927(7) | 49.41% |
| 8.9Kb | incomplete | 20 | 9 | 64025-73006 | PHAGE_Achrom_Motura_NC_049849(2) | 57.48% |
| **510290- *Cronobacter spp.*** | | | | | | |
| 24.7Kb | intact | 150 | 36 | 158-24952 | PHAGE_Salmon_118970_sal3_NC_031940(11) | 51.75% |
| 14.3Kb | incomplete | 20 | 8 | 28729-43127 | PHAGE_Cronob_phiES15_NC_018454(3) | 52.21% |
| 7.2Kb | incomplete | 20 | 9 | 39-7302 | PHAGE_Erwini_vB_EhrS_59_NC_048198(5) | 51.93% |
| 41.7Kb | intact | 150 | 50 | 1108-42836 | PHAGE_Salmon_SEN8_NC_047753(26) | 53.06% |
| 16Kb | incomplete | 30 | 12 | 2354-18367 | PHAGE_Cronob_ENT47670_NC_019927(9) | 48.09% |
| 10.2Kb | incomplete | 60 | 12 | 150803-161046 | PHAGE_Acinet_vB_AbaM_ME3_NC_041884(3) | 49.76% |
| 8.9Kb | incomplete | 20 | 9 | 159874-168855 | PHAGE_Achrom_Motura_NC_049849(2) | 57.48% |
| **510199- *Cronobacter spp.*** | | | | | | |
| 7.2Kb | incomplete | 20 | 9 | 518490-525753 | PHAGE_Erwini_vB_EhrS_49_NC_048197(5) | 51.93% |
| 24.7Kb | intact | 150 | 36 | 158-24952 | PHAGE_Salmon_118970_sal3_NC_031940(11) | 51.75% |
| 14.3Kb | incomplete | 20 | 9 | 28729-43127 | PHAGE_Cronob_phiES15_NC_018454(3) | 52.21% |
| 10.2Kb | incomplete | 60 | 13 | 248668-258911 | PHAGE_Acinet_vB_AbaM_ME3_NC_041884(3) | 49.76% |
| 29.4Kb | incomplete | 30 | 10 | 314140-343626 | PHAGE_Salmon_118970_sal3_NC_031940(2) | 53.66% |
| 16.5Kb | incomplete | 20 | 11 | 341-16901 | PHAGE_Cronob_ESSI_2_NC_047854(9) | 50.96% |
| 38.1Kb | intact | 150 | 47 | 268314-306501 | PHAGE_Salmon_SEN8_NC_047753(26) | 53.15% |
| 16Kb | incomplete | 30 | 12 | 262047-278060 | PHAGE_Cronob_ENT47670_NC_019927(9) | 48.09% |
| 8.9Kb | incomplete | 20 | 9 | 159874-168855 | PHAGE_Achrom_Motura_NC_049849(2) | 57.48% |
| **510557- *Cronobacter spp.*** | | | | | | |
| 49.1Kb | intact | 150 | 70 | 84454-133636 | PHAGE_Cronob_ENT47670_NC_019927(20) | 50.68% |
| 8.4Kb | incomplete | 10 | 8 | 306-8748 | PHAGE_Klebsi_ST13_OXA48phi12.1_NC_049453(5) | 52.86% |
| 13.5Kb | incomplete | 20 | 14 | 196283-209782 | PHAGE_Prochl_P_TIM68_NC_028955(2) | 53.99% |
| 9.1Kb | incomplete | 30 | 9 | 122987-132156 | PHAGE_Mycoba_Bactobuster_NC_031279(1) | 58.06% |
| 8.6Kb | incomplete | 20 | 9 | 145266-153931 | PHAGE_Bacill_G_NC_023719(2) | 59.44% |
| 8.9Kb | incomplete | 20 | 9 | 135958-144953 | PHAGE_Achrom_Motura_NC_049849(2) | 57.65% |
| 8.8Kb | incomplete | 20 | 10 | 39494-48307 | PHAGE_Agroba_Atu_ph07_NC_042013(2) | 51.60% |
| **510556- *Cronobacter spp.*** | | | | | | |
| 8.6Kb | incomplete | 20 | 8 | 139200-147863 | PHAGE_Bacill_G_NC_023719(2) | 59.28% |
| 6.3Kb | incomplete | 10 | 7 | 7478-13845 | PHAGE_Bacill_G_NC_023719(3) | 57.73% |
| 31.7Kb | intact | 94 | 39 | 95387-127113 | PHAGE_Cronob_ESSI_2_NC_047854(27) | 52.95% |
| **510535- *Salmonella spp.*** | | | | | | |
| 5.1Kb | incomplete | 20 | 9 | 395174-400301 | PHAGE_Mannhe_vB_MhM_1127AP1_NC_047750(2) | 51.05% |
| 28.5Kb | incomplete | 60 | 19 | 15652-44172 | PHAGE_Escher_500465_1_NC_049342(12) | 54.81% |
| 8.4Kb | incomplete | 20 | 9 | 123747-132148 | PHAGE_Escher_500465_2_NC_049343(5) | 53.37% |
| 20.4Kb | incomplete | 40 | 30 | 18419-38838 | PHAGE_Burkho_phiE255_NC_009237(16) | 50.57% |
| 14.2Kb | incomplete | 50 | 19 | 163091-177347 | PHAGE_Salmon_118970_sal3_NC_031940(5) | 47.50% |
| **510536- *Salmonella spp.*** | | | | | | |
| 6.2Kb | incomplete | 20 | 6 | 3703-9996 | PHAGE_Erwini_Hena1_NC_048828(3) | 53.18% |
| **510537- *Salmonella spp.*** | | | | | | |
| 55.2Kb | intact | 150 | 67 | 30177-85444 | PHAGE_Salmon_118970_sal3_NC_031940(66) | 50.38% |
| 10.5Kb | incomplete | 10 | 10 | 432-10937 | PHAGE_Entero_phi92_NC_023693(5) | 45.45% |
| 6.2Kb | incomplete | 20 | 6 | 53673-59966 | PHAGE_Erwini_Hena1_NC_048828(3) | 53.18% |
| **510538- *Salmonella spp.*** | | | | | | |
| 6.3Kb | incomplete | 20 | 6 | 40543-46896 | PHAGE_Erwini_pEp_SNUABM_01_NC_048807(3) | 53.13% |
| 8.3Kb | incomplete | 20 | 8 | 141967-150315 | PHAGE_Escher_RCS47_NC_042128(3) | 57.26% |
| 8.4Kb | incomplete | 20 | 9 | 104129-112530 | PHAGE_Escher_500465_2_NC_049343(5) | 53.37% |
| 20.4Kb | incomplete | 40 | 30 | 157656-178075 | PHAGE_Burkho_phiE255_NC_009237(16) | 50.57% |
| 14.2Kb | incomplete | 50 | 19 | 164146-178402 | PHAGE_Salmon_118970_sal3_NC_031940(5) | 47.50% |
| **510539- *Salmonella spp.*** | | | | | | |
| 5.1Kb | incomplete | 20 | 8 | 333836-338963 | PHAGE_Mannhe_vB_MhM_1127AP1_NC_047750(2) | 51.05% |
| 8.4Kb | incomplete | 20 | 9 | 87353-95754 | PHAGE_Escher_500465_2_NC_049343(5) | 53.37% |
| 14.2Kb | incomplete | 50 | 19 | 2487-16743 | PHAGE_Salmon_118970_sal3_NC_031940(5) | 47.50% |
| 55.2Kb | intact | 150 | 65 | 32818-88085 | PHAGE_Salmon_118970_sal3_NC_031940(66) | 50.38% |
| **510540- *Salmonella spp.*** | | | | | | |
| 20.4Kb | incomplete | 40 | 30 | 18419-38838 | PHAGE_Burkho_BcepMu_NC_005882(16) | 50.57% |
| 5.1Kb | incomplete | 20 | 8 | 329687-334814 | PHAGE_Mannhe_vB_MhM_1127AP1_NC_047750(2) | 51.05% |
| 8.4Kb | incomplete | 20 | 9 | 87352-95753 | PHAGE_Escher_500465_2_NC_049343(5) | 53.37% |
| 6.3Kb | incomplete | 20 | 6 | 36223-42576 | PHAGE_Erwini_pEp_SNUABM_01_NC_048807(3) | 53.13% |
| 8.3Kb | incomplete | 20 | 8 | 137647-145995 | PHAGE_Escher_RCS47_NC_042128(3) | 57.26% |
| 12.2Kb | incomplete | 40 | 14 | 4971-17261 | PHAGE_Salmon_118970_sal3_NC_031940(5) | 47.02% |
| 11.4Kb | incomplete | 10 | 11 | 166792-178224 | PHAGE_Entero_phi92_NC_023693(5) | 44.36% |
